# Oscillatory Cortical Activity in an Animal Model of Dystonia Caused by Cerebellar Dysfunction

**DOI:** 10.3389/fncel.2018.00390

**Published:** 2018-11-06

**Authors:** Elena Laura Georgescu, Ioana Antoaneta Georgescu, Carmen Denise Mihaela Zahiu, Alexandru Răzvan Şteopoaie, Vlad Petru Morozan, Adrian Ştefan Pană, Ana-Maria Zăgrean, Daniela Popa

**Affiliations:** ^1^Division of Physiology and Neuroscience, Carol Davila University of Medicine and Pharmacy, Bucharest, Romania; ^2^Institut de Biologie de l’Ecole Normale Supérieure (IBENS), Ecole Normale Supérieure, CNRS, INSERM, PSL Research University, Paris, France

**Keywords:** cerebellum, intra-cortical oscillations, dystonia, kainate, mice

## Abstract

The synchronization of neuronal activity in the sensorimotor cortices is crucial for motor control and learning. This synchrony can be modulated by upstream activity in the cerebello-cortical network. However, many questions remain over the details of how the cerebral cortex and the cerebellum communicate. Therefore, our aim is to study the contribution of the cerebellum to oscillatory brain activity, in particular in the case of dystonia, a severely disabling motor disease associated with altered sensorimotor coupling. We used a kainic-induced dystonia model to evaluate cerebral cortical oscillatory activity and connectivity during dystonic episodes. We performed microinjections of low doses of kainic acid into the cerebellar vermis in mice and examined activities in somatosensory, motor and parietal cortices. We showed that repeated applications of kainic acid into the cerebellar vermis, for five consecutive days, generate reproducible dystonic motor behavior. No epileptiform activity was recorded on electrocorticogram (ECoG) during the dystonic postures or movements. We investigated the ECoG power spectral density and coherence between motor cortex, somatosensory and parietal cortices before and during dystonic attacks. During the baseline condition, we found a phenomenon of permanent adaptation with a change of baseline locomotor activity coupled to an ECoG gamma band increase in all cortices. In addition, after kainate administration, we observed an increase in muscular activity, but less signs of dystonia together with modulations of the ECoG power spectra with an increase in gamma band in motor, parietal and somatosensory cortices. Moreover, we found reduced coherence in all measured frequency bands between the motor cortex and somatosensory or parietal cortices compared to baseline. In conclusion, examination of cortical oscillatory activities in this animal model of chronic dystonia caused by cerebellar dysfunction reveals a disruption in the coordination of neuronal activity across the cortical sensorimotor/parietal network, which may underlie motor skill deficits.

## Introduction

There is increasing evidence that oscillations in the sensorimotor cortices can be modulated by the cerebellum via cerebello-thalamo-cortical pathways. The cerebellum provides a putative synchronization mechanism across multiple regions of the brain in both rodents and humans (O’Connor et al., 2002; Kujala et al., 2007; Courtemanche et al., 2013; Popa et al., 2013). Yet, the role of the cerebellum in modulating cerebral oscillations and associated coherence between brain regions involved in motor execution remains poorly understood.

Dystonia is a motor disorder in which a cerebellar dysfunction has been recently recognized, despite the absence of cardinal cerebellar signs (ataxia, dysmetria). Abnormal oscillatory activities in the motor cortex and abnormal learning are recognized as dystonic typical traits. This has led to propose that a distorted cerebellar output in the cerebello-thalamo-cortical pathway may pathologically influence the motor cortex (Prudente et al., 2014). Results of recent animal studies corroborate such a view (Caligiore et al., 2017). Indeed, abnormalities restricted to cerebellum were sufficient to cause dystonia and the cerebellar dysfunction was coupled to dystonic movements (LeDoux, 2011; Raike et al., 2013; Fremont et al., 2014; White and Sillitoe, 2017).

So far, studies in dystonia have focused on cortical oscillations during simple movements and did not provide information on cerebellar contribution to these oscillations. Dystonic patients have impaired movement-related beta band coherence during simple movements in primary sensorimotor cortices (Jin et al., 2011). In focal hand dystonia patients, a significant decrease in high gamma power in the sensorimotor cortex was identified during the preparation of simple movements of the affected hand, when compared to healthy subjects (Hinkley et al., 2012). Furthermore, recent studies provide evidence of reduced functional connectivity in theta, alpha and beta bands in the somatosensory network in patients with Writer’s Cramp dystonia (Cheng et al., 2016).

Dystonia can be pharmacologically modeled in mice by direct application of a glutamate receptor agonist (kainic acid) on the cerebellar cortex. In this case, abnormal cerebellar output is the source of dystonia (Pizoli et al., 2002). When dystonic movements were triggered by pharmacological stimulation of the cerebellum, microdialysis revealed significant reductions in striatal dopamine release. These results suggest that dystonia may originate from the alteration of a motor network involving both the basal ganglia and the cerebellum (Chen et al., 2014; Neumann et al., 2015, 2017), rather than an isolated dysfunction of only these motor areas.

Structural changes of white matter connectivity between the red nucleus and internal pallidum in the basal ganglia have been described in dystonic patients (Blood et al., 2012; Blood, 2013). The neurons from magnocellular red nucleus receive excitatory input from the contralateral cerebellar nuclei (dentate and interposed) and release output through the descending rubrospinal tract that sends the information to the interneurons of the ventral gray column that synapse with the contralateral motoneurons (Fedina et al., 1975). Because the motoneurons also receive input directly from the rubrospinal axons, they will be activated both through the rubrospinal tract (directly) and also through the propriospinal neurons (indirectly). Thus, an abnormal cerebellar output may cause a deficient agonist and antagonist muscle coordination which occurs in dystonia (Pizoli et al., 2002). Abnormal cerebellar output coupled with dystonia-like behavior was also induced in mice by blocking the glutamatergic olivocerebellar signaling and eliminating the Purkinje cell complex spikes activity. In addition, *in vivo* lidocaine infusions in the cerebellar nuclei of these mice reduced dystonic tremor. Also, deep brain stimulation of the interposed cerebellar nuclei improved movement in severely dystonic mice (White and Sillitoe, 2017).

Abnormal activities in another cortical area, the parietal cortex, were also described in dystonic patients (Gallea et al., 2016). Intricate sensory maps for the planning of eye or hand reaching movements represent the form in which the parietal cortex is involved in creating cognitive plans. Each movement is individually represented on the parietal intentional map that serves as an integrator of various sensory inputs and as a coordination area. Also, the parietal cortex undergoes rapid plastic and interpersonal variations (Andersen and Buneo, 2002).

The present study examined the contribution of the cerebellum to motor, somatosensory and parietal oscillatory activities by using a mouse model of dystonia (chronic kainic acid administration to the cerebellar vermis). Kainic acid is an excitatory glutamate agonist proven to induce generalized dystonia when injected into the cerebellar vermis (Pizoli et al., 2002). We combined *in vivo* recordings of motor behavior, electrocorticogram (ECoG) and electromyogram (EMG) in order to characterize oscillatory activity in somatosensory, motor and parietal cortices during five consecutive days of sustained dystonic motor behavior. Moreover, our multi-site recording technique allowed us to calculate the coherence between motor cortex and somatosensory cortex, or between motor and parietal cortices, before and during dystonic attacks. Coherence describes the spectral distribution of oscillatory synchronization between simultaneously recorded signals and may reflect interactions or communication between brain areas or as areas sharing a common drive (Bowyer, 2016).

## Materials and Methods

The study was carried out with the approval of the local committee (Comisia de Etică a Cercetării Ştiinţifice; number PO-35-F-01) for animal research of “Carol Davila” University of Medicine and Pharmacy (Bucharest, Romania). The European Communities Council Directive 86/609/EEC and national policies for the good practice on animals used for scientific purposes were respected.

### Animals

Experiments were performed on 3-month-old Swiss albino mice (*n* = 20), divided into two groups, motor-somatosensory group (*n* = 10) and motor-parietal group (*n* = 10), weighing between 45 g and 52 g. Mice were provided access to water and food *ad libitum* and housed on a 12 h light/dark cycle.

### Chronic Electrode Implantation Surgery

**Figure 1:**
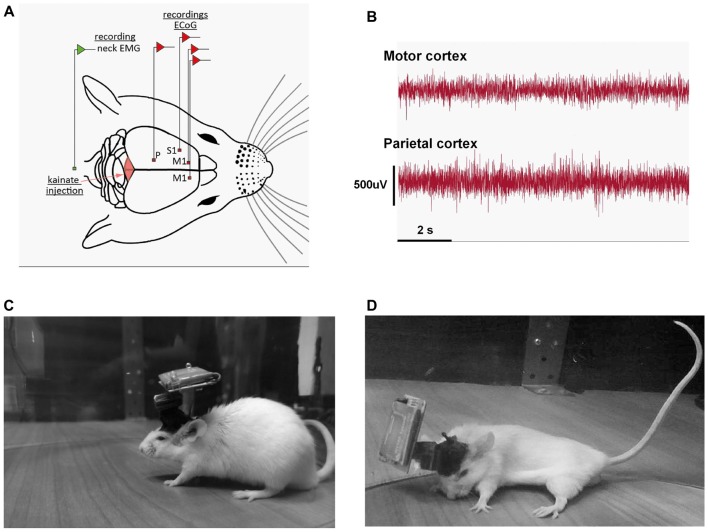
**(A)** Schematic representation of the recording conditions; example of the position of the electrodes for the motor (M1), somatosensory (S1) and parietal (P) group. **(B)** Example of a motor, parietal electrocorticograms (ECoGs) during a dystonic attack. **(C)** Example of recording of a mouse in the pre-kainate state. **(D)** Example of dystonic attack during a post-kainate recording session.

Inhalatory isoflurane anesthesia (3%–4% concentration) was used for induction and 1.5%–2% for maintenance together with buprenorphine (50 μg/kg) applied subcutaneously for pain management. During surgical preparation, anesthetic efficiency was assessed by checking the withdrawal reflex to a noxious stimulus; if necessary, isoflurane dose was increased. Mice were maintained at 37°C through the entire procedure. After a local subcutaneous anesthetic injection (lidocaine, 1 ml, 2%), the scalp was incised medially, and skin and subcutaneous tissue were removed from the skull. Four small (1 mm diameter) craniotomies were drilled with stereotaxic guidance for the insertion of in-house made insulated nichrome (Kanthal, Palm Coast, FL, USA), flexible wire (0.15 mm) ECoG electrodes, on the dura mater surface. All the electrodes were manually de-insulated at both ends, 2 mm each, by mechanical abrasion. The two groups of implanted mice differed only by the position of the somatosensory or the parietal electrode. The placement of the electrodes was above both the left and the right motor cortices (2.2 mm anterior and 2.2 mm lateral relative to Bregma) and the ground electrode at 2 mm posterior and 2 mm lateral to the right relative to lambda. An EMG electrode, made from the same wire, was placed in the neck muscles. For the somatosensory cortex group another electrode was placed above the left somatosensory cortex (1.3 mm anterior and 3.2 mm lateral relative to the Bregma) and, for the parietal cortex group, the electrode was fixed at 2.06 mm posterior and 2.3 mm lateral relative to the Bregma (Figure 1A). The skull was then coated with Super Bond (Dental Adhesive Resin Cement, Sun Medical CO, Japan). The electrodes were then fixed with dental cement (Pi-Ku-Plast HP 36, Bredent GmbH, Germany) and connected to the pins through which the headstage would be attached. At 7 mm posterior to Bregma, on the cerebellar vermal lobule VI, we inserted on the surface of the dura mater a guide cannula vertically with a 0.6 mm internal diameter. After the surgery, another dose of buprenorphine (50 μg/kg) was applied. The mice were individually housed and were allowed a minimum of 4 days of recovery after implantation.

### Data Acquisition

Recordings (ECoG along with EMG activity) were performed in awake, freely moving animals for 150 min for six consecutive days using a Multi Channel Systems W2100 wireless interface board with an acquisition frequency of 1 KHz and a 4-channel W2100-HS4-opto Headstage (weight of 1.9 g + 3.8 g for the battery). In addition, video tracking of the mice was carried out for all experiments. After surgery, all mice were kept in the same room where the recordings followed. We also habituated the animals to the recording arena 2 days before the recordings. In the first day of experiment (baseline day), no injection was performed. On the next five consecutive days, recordings were performed before (10 min) and after 150 (min) kainic acid (Sigma) injections. Mice were briefly anesthetized with isoflurane (SC. Rompharm Company S.R.L., Romania) for the kainate injection and, after 1 min from waking up, the recording started. The ECoG demonstrated the absence of epileptic seizures after the injection.

### Data Analysis

We only included data acquired from confirmed recording sites. All data were visually inspected, and intervals contaminated by artifacts were excluded from further analysis. Consequently, an average of 80% of recordings was retained. Power spectral density estimates were obtained with the Welch method with 2 s windows overlapping 1 s from the 1 KHz signal (MATLAB function *pwelch*). Changes in the ECoG power spectral density bands (Delta: 0.5–3.5 Hz, Theta: 4–12 Hz; Beta: 13–30 Hz; Low-Gamma: 30.5–48 Hz; High-Gamma: 52–100 Hz) were analyzed before and during all days of kainic acid administration for all the areas examined: motor, somatosensory and parietal cortices. Moreover, the real and imaginary coherence variations per band were investigated, as well as the evolution in time throughout the 5 days of chronic application of kainic acid for all the investigated brain regions. *Coherence* estimates were obtained using the MATLAB function *mscohere*, from power spectral density estimates of pairs of ECoG recordings, using the same parameters investigating the coherence between motor and sensorimotor and between motor and parietal cortices. The imaginary part of the coherence was also computed to estimate the coherence avoiding contamination by volume conduction (Nolte et al., 2004). Data were normalized by expressing the results as percentages of the baseline values (the values from the first day of recording).

The data obtained were processed in the Excel 2016 software, and then in GraphPrism 6.0 using repeated measures ANOVA, Dunnett’s multiple comparisons test, Friedman test, Dunn’s multiple comparisons test, Multiple *t*-test, Mann Whitney test and Kruskal-Wallis test after testing the normality distribution of the data, as appropriate. Results were presented as mean ± the standard error of the mean (SEM). A *p*-value < 0.05 was considered statistically significant.

Electromyography analysis consisted of first calculating the power spectral density for each EMG recording for the whole 1–100-Hz frequency range. To quantitatively compare the EMG spectra for the two functional states, pre-kainate and post-kainate, mean power frequency was calculated. In addition, the median frequency was calculated as the frequency at which the EMG power spectrum is divided into two regions with equal amplitude. We also analyzed the EMG amplitude estimators: root mean square (RMS) and average rectified value (ARV; μV). To investigate the effects on the EMG spectral power and amplitude, the analysis consisted of ANOVA with repeated measures.

### Video Recordings Monitored Behavior and Focused on the Animals’ Motor Behavior in an Open Field

We induced dystonia using administration of 0.75 ± 0.1 μl (100 μg/ml) of kainate directly on the vermis surface of the cerebellum (Pizoli et al., 2002). The first day of recordings (without injection) was considered the baseline for each mouse. Periods of paroxysmal dystonic attacks were identified offline based upon the neck EMG recordings and video recordings. The presence and severity of dystonia in mice was quantified using a previously published scale (Pizoli et al., 2002; Calderon et al., 2011) in which 0 = normal motor behavior; 1 = abnormal motor behavior, no dystonic postures; 2 = mild motor impairment, dystonic-like postures when disturbed; 3 = moderate impairment, frequent spontaneous dystonic postures; and 4 = severe impairment, sustained dystonic postures. The percentage rate of active wake behavior from the total time of a recording (active wake percentages, AW%) was also calculated for both states, pre and post-kainate. In order to assess the general locomotor activity, we considered active wake behavior as the exploring activity during which the mice were walking inside the open field. Dystonia severity was evaluated for each 10-min epoch and several reviewers performed the assessment of the behavior independently (DZ, AŞ, VM and AP). The reviewers were blind to the procedures that were done, and their scores were averaged and decoded.

### Correlations Between Behavior and Neuronal Activity

We assessed the link between behavior, dystonia and neuronal activity by computing the correlations between dystonic behavior (dystonia score or active wake) and the ECoG coherence for both motor cortex-somatosensory cortex and motor cortex-parietal cortex. Linear regressions were analyzed and Pearson correlation coefficients (*r*) and significance (*P*) values were added to Supplementary Tables.

## Results

### Mice Displayed Dystonic Motor Behavior After Cerebellar Kainic Acid Application on the Cerebellar Vermis

Wild-type mice displayed a reproducible dystonic behavior after kainate injection on the lobule VI of the cerebellar vermis (Figures 1A–D). The produced phenotype was similar to that previously described (Pizoli et al., 2002).

ECoG (motor, somatosensory, parietal) recordings during dystonic attacks indicated no epileptiform activity (Figure 1B). The first signs of dystonia appeared after ~2–3 min following the injection with a general slowing down of movements or the hind limb being held near the trunk while the mouse was exploring. After a few minutes, the mice began to show attacks of generalized dystonia with the muscles of the trunk, neck, tail and limbs being contracted (arched back, flexed neck, tail held in an upright position; Figure 1D). Mice usually remained in an immobilized severe dystonic position for a few minutes, followed by periods of lessening of the symptoms.

**Figure 2:**
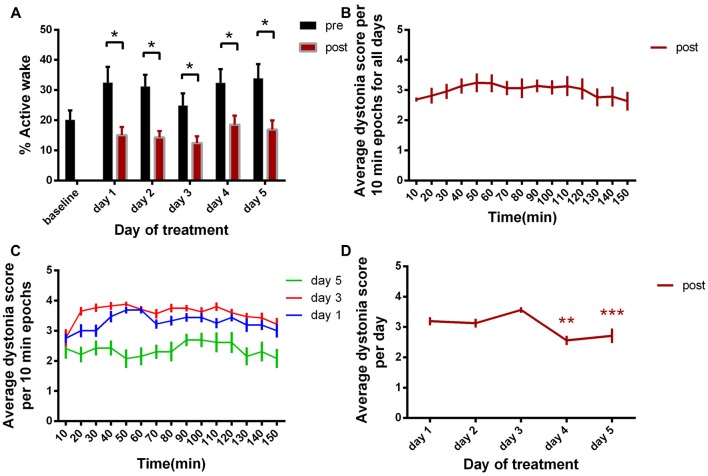
**(A)** Active wake percentage averages ± standard error of the mean (SEM) from baseline day to day 5 evolution. **(B)** Average score of dystonia during the recording sessions ± SEM. **(C)** Average dystonia score during day 1, day 3, day 5. **(D)** Average total dystonia score per day ± SEM evolution from day 1 to day 5. Results are expressed as average ± the SEM (*p < 0.05, **p < 0.01, ***p < 0.001, Table 1).

When comparing baseline day with all pre-kainate and post-kainate behavior of the same mice, we found that the mice were less active in post-kainate states (Figure 2A, Table 1). We also observed differences between the AW% during the pre-administration periods across all 5 days of dystonia. These results showed a sustained decrease in the time spent in exploratory activity after kainic injections. Interestingly, we also found an increase of the AW% before the injection across days (Figure 2A, Table 1), suggesting the possible presence of long-lasting plastic changes in the motor system following recurring kainate injections.

**Table 1:** Behavior (active wake percentages, AW%).

Behavior (Figure 2A)	Test		*P* value
AW% baseline vs. AW% post day1	Multiple *t*-test		*P* = 0.0044
AW% baseline vs. AW% post day2	Multiple *t*-test		*P* = 0.0004
AW% baseline vs. AW% post day3	Multiple *t*-test		*P* = 0.0123
AW% baseline vs. AW% post day4	Multiple *t*-test		*P* = 0.0140
AW% baseline vs. AW% post day5	Multiple *t*-test		*P* = 0.0080
**Dystonia score (Figure 2D)**	Friedman test		*P* < 0.0001
day1 vs. day2	+Dunn’s multiple comparisons test		ns
day1 vs. day3	+Dunn’s multiple comparisons test		ns
day1 vs. day4	+Dunn’s multiple comparisons test		**
day1 vs. day5	+Dunn’s multiple comparisons test		***
**EMG median/mean frequency (Figures 3B,C)**	1-way ANOVA	*F*_(1.134,226.7)_ = 9.025	*P* = 0.0020
day0 vs. day1	Dunnett’s multiple comparisons test		***
day0 vs. day2	Dunnett’s multiple comparisons test		ns
day0 vs. day3	Dunnett’s multiple comparisons test		**
day0 vs. day4	Dunnett’s multiple comparisons test		**
day0 vs. day5	Dunnett’s multiple comparisons test		*
**RMS EMG (Figure 3E)**	Kruskal-Wallis test		*P* = 0.6832
day0 vs. day1	Dunn’s multiple comparisons test		ns
day0 vs. day2	Dunn’s multiple comparisons test		ns
day0 vs. day3	Dunn’s multiple comparisons test		ns
day0 vs. day4	Dunn’s multiple comparisons test		ns
day0 vs. day5	Dunn’s multiple comparisons test		ns
**ARV EMG (Figure 3F)**	Kruskal-Wallis test		*P* = 0.9260
day0 vs. day1	Dunn’s multiple comparisons test		ns
day0 vs. day2	Dunn’s multiple comparisons test		ns
day0 vs. day3	Dunn’s multiple comparisons test		ns
day0 vs. day4	Dunn’s multiple comparisons test		ns
day0 vs. day5	Dunn’s multiple comparisons test		ns

Electromyogram (EMG) median and mean frequency. Statistical data. *p < 0.05, **p < 0.01, ***p < 0.001, ns, not significant.

### Evaluation of Dystonia Score

We then examined the average dystonia score for every 10 min recording periods (Figure 2B, Table 1). The results showed a high score over the complete duration of the recording, with a peak at approximatively 50 min from the recording start. This progress was followed by descending scores, constantly until the end of the recording (150 min). Figure 2C represents the evolution of the average dystonia score from the beginning to the end of the recording during day 1, 3 and 5 of kainic acid administration that shows a maximum of scores during day 3 and a minimum during day 5. Moreover, we analyzed the variation over time of the average score of these changes for the five consecutive days. When comparing each day to day 1, we found statistically significant decreased total average scores on day 4 and day 5 of kainic acid administration (Figure 2D, Table 1). The findings also implied that the severity of dystonia increased until day 3 and afterwards it started to decrease significantly to a minimum on the last day of the experiment, day 5, demonstrating that the susceptibility to kainate was possibly reduced at the end, suggesting a compensation mechanism or receptor desensitization.

### Electromyography Demonstrates Higher Muscular Activity After Cerebellar Kainate Injections

**Figure 3:**
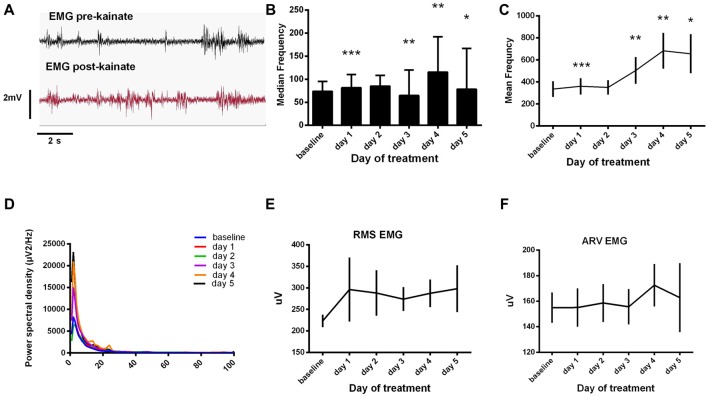
**(A)** Raw electromyogram (EMG) recordings. Examples of raw EMG recordings during the dystonic attack. **(B)** Median frequency of the EMG power spectra ± SEM. **(C)** Mean frequency of the EMG power spectra ± SEM. **(D)** Average EMG power spectral density evolution from baseline day to day 5. **(E)** Average root mean square (RMS) during each day of experiment (μV). **(F)** Average rectified value (ARV) during all days of experiment (μV). **(B,C)** Repeated measures ANOVA, Dunnett’s multiple comparisons test, each day vs. baseline day, Table 1. *p < 0.05, **p < 0.01, ***p < 0.001.

During the dystonic attacks, mice had a predominantly tonic pattern of muscle activity (Figure 3A). We examined the electromyographic recordings and we found that the median frequency of the muscular power spectral density was increased in day 1, day 4 and day 5 of experiment and decreased in day 3 (Figure 3B). The values recorded a maximum on day 4 and, in day 5, they fell again below the previous day ones. The mean frequency had a continuous increase since day 1 until day 4 of recordings and started to decrease in day 5 (Figure 3C, Table 1). Still, the values were higher than in the baseline condition. After the kainic acid injection, the power spectral density peak amplitude (Figure 3D) increased gradually from the baseline day until day 5.

We further analyzed the EMG amplitude estimators (Figures 3E,F). RMS revealed high amplitudes in the first and last days of kainate administration and minimum ones in day 3. The ARV estimator showed minimum changes in the first 3 days, recorded a maximum in day 4 and dropped immediately in the 5th day of experiment. The values recorded in day 5 were still higher than those recorded in the first 3 days. However, these changes were not statistically significant. This data suggests that kainic acid still has a muscular effect (increased mean frequency) during the last days of recording, even though dystonic score decreases.

#### Motor Cortex ECoG Power Spectral Density Evolution

**Figure 4:**
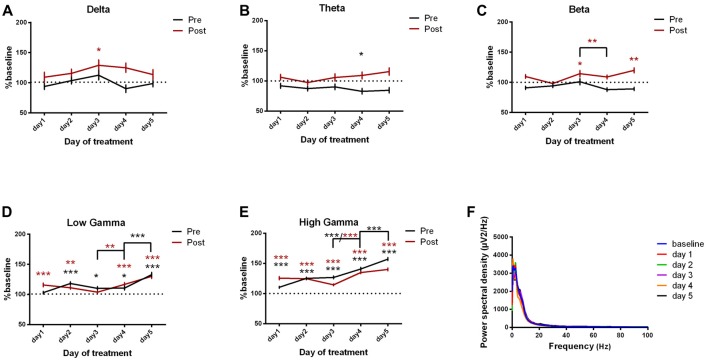
Power spectral density of motor cortex ECoGs represented as percentage from baseline day across different bands. **(A)** Delta (0.5–3.5 Hz). **(B)** Theta (4–12 Hz). **(C)** Beta (13 -30 Hz). **(D)** Low-gamma (30.5–48 Hz). **(E)** High-gamma (52–100 Hz) oscillations from baseline day to day 1, day 2, day 3, day 4, day 5 of intracerebellar kainic acid administration. **(F)** Average ECoG power spectral density evolution from baseline day to the 5th day. For each frequency band, the spectra are expressed as average ± the SEM (*p < 0.05, **p < 0.01, ***p < 0.001, repeated measures ANOVA, Dunnett’s multiple comparisons test, each day vs. baseline day with the values of baseline group being normalized as 100%, Table 2).

For the motor cortex, we found significant power spectral density increases in the high frequency bands, especially in low and high gamma bands, for both pre and post-kainate recordings. Here, changes were visible since the first day of kainic acid administration; significant changes were also found in low frequency bands (Figures 4A–E, Table 2).

**Table 2:** Motor cortex power spectral density bands.

Motor cortex spectral density (Figure 4)	Test	*F*_(DFn, DFd)_	*P* value
Delta pre	1-way ANOVA	*F*_(5,701)_ = 1.558	*P* = 0.1698
day0 vs. day1	+Dunnett’s multiple comparisons test		ns
day0 vs. day2	+Dunnett’s multiple comparisons test		ns
day0 vs. day3	+Dunnett’s multiple comparisons test		ns
day0 vs. day4	+Dunnett’s multiple comparisons test		ns
day0 vs. day5	+Dunnett’s multiple comparisons test		ns
Delta post	1-way ANOVA	*F*_(5,694)_ = 1.982	*P* = 0.0792
day0 vs. day1	+Dunnett’s multiple comparisons test		ns
day0 vs. day2	+Dunnett’s multiple comparisons test		ns
day0 vs. day3	+Dunnett’s multiple comparisons test		*
day0 vs. day4	+Dunnett’s multiple comparisons test		ns
day0 vs. day5	+Dunnett’s multiple comparisons test		ns
Theta pre	1-way ANOVA	*F*_(5,1711)_ = 1.947	*P* = 0.0838
day0 vs. day1	+Dunnett’s multiple comparisons test		ns
day0 vs. day2	+Dunnett’s multiple comparisons test		ns
day0 vs. day3	+Dunnett’s multiple comparisons test		ns
day0 vs. day4	+Dunnett’s multiple comparisons test		*
day0 vs. day5	+Dunnett’s multiple comparisons test		ns
Theta post	1-way ANOVA	*F*_(5,1694)_ = 1.481	*P* = 0.1930
day0 vs. day1	+Dunnett’s multiple comparisons test		ns
day0 vs. day2	+Dunnett’s multiple comparisons test		ns
day0 vs. day3	+Dunnett’s multiple comparisons test		ns
day0 vs. day4	+Dunnett’s multiple comparisons test		ns
day0 vs. day5	+Dunnett’s multiple comparisons test		ns
Beta pre	1-way ANOVA	*F*_(5,3529)_ = 2.536	*P* = 0.0267
day0 vs. day1	+Dunnett’s multiple comparisons test		ns
day0 vs. day2	+Dunnett’s multiple comparisons test		ns
day0 vs. day3	+Dunnett’s multiple comparisons test		ns
day0 vs. day4	+Dunnett’s multiple comparisons test		ns
day0 vs. day5	+Dunnett’s multiple comparisons test		ns
Beta post	1-way ANOVA	*F*_(5,3494)_ = 5.058	*P* = 0.0001
day0 vs. day1	+Dunnett’s multiple comparisons test		ns
day0 vs. day2	+Dunnett’s multiple comparisons test		ns
day0 vs. day3	+Dunnett’s multiple comparisons test		*
day0 vs. day4	+Dunnett’s multiple comparisons test		ns
day0 vs. day5	+Dunnett’s multiple comparisons test		**
Low gamma pre	1-way ANOVA	*F*_(5,3630)_ = 17.78	*P* < 0.0001
day0 vs. day1	+Dunnett’s multiple comparisons test		ns
day0 vs. day2	+Dunnett’s multiple comparisons test		***
day0 vs. day3	+Dunnett’s multiple comparisons test		*
day0 vs. day4	+Dunnett’s multiple comparisons test		*
day0 vs. day5	+Dunnett’s multiple comparisons test		***
Low gamma post	1-way ANOVA	*F*_(5,3594)_ = 13.56	*P* < 0.0001
day0 vs. day1	+Dunnett’s multiple comparisons test		***
day0 vs. day2	+Dunnett’s multiple comparisons test		**
day0 vs. day3	+Dunnett’s multiple comparisons test		ns
day0 vs. day4	+Dunnett’s multiple comparisons test		***
day0 vs. day5	+Dunnett’s multiple comparisons test		***
High gamma pre	1-way ANOVA	*F*_(5,9791)_ = 100.7	*P* < 0.0001
day0 vs. day1	+Dunnett’s multiple comparisons test		***
day0 vs. day2	+Dunnett’s multiple comparisons test		***
day0 vs. day3	+Dunnett’s multiple comparisons test		***
day0 vs. day4	+Dunnett’s multiple comparisons test		***
day0 vs. day5	+Dunnett’s multiple comparisons test		***
High gamma post	1-way ANOVA	*F*_(5,9694)_ = 45.23	*P* < 0.0001
day0 vs. day1	+Dunnett’s multiple comparisons test		***
day0 vs. day2	+Dunnett’s multiple comparisons test		***
day0 vs. day3	+Dunnett’s multiple comparisons test		***
day0 vs. day4	+Dunnett’s multiple comparisons test		***
day0 vs. day5	+Dunnett’s multiple comparisons test		***
Delta pre			
day3 vs. day4	Mann Whitney test		*P* = 0.1239
day4 vs. day5	Mann Whitney test		*P* = 0.1975
Delta post			
day3 vs. day4	Mann Whitney test		*P* = 0.8639
day4 vs. day5	Mann Whitney test		*P* = 0.2458
Theta pre			
day3 vs. day4	Mann Whitney test		*P* = 0.4611
day4 vs. day5	Mann Whitney test		*P* = 0.8121
Theta post			
day3 vs. day4	Mann Whitney test		*P* = 0.1490
day4 vs. day5	Mann Whitney test		*P* = 0.4406
Beta pre			
day3 vs. day4	Mann Whitney test		*P* = 0.5772
day4 vs. day5	Mann Whitney test		*P* = 0.1614
Beta post			
day3 vs. day4	Mann Whitney test		*P* = 0.0023
day4 vs. day5	Mann Whitney test		*P* = 0.1052
Low gamma pre			
day3 vs. day4	Mann Whitney test		*P* = 0.6894
day4 vs. day5	Mann Whitney test		*P* < 0.0001
Low gamma post			
day3 vs. day4	Mann Whitney test		*P* = 0.0037
day4 vs. day5	Mann Whitney test		*P* = 0.1339
High gamma pre			
day3 vs. day4	Mann Whitney test		*P* < 0.0001
day4 vs. day5	Mann Whitney test		*P* < 0.0001
High gamma post			
day3 vs. day4	Mann Whitney test		*P* < 0.0001
day4 vs. day5	Mann Whitney test		*P* = 0.4010

Statistical data. *p < 0.05, **p < 0.01, ***p < 0.001, ns, not significant.

Overall, except for delta band, all motor cortex ECoG power spectral densities bands showed a gradual increase that reached its maximum in the 5th day of examination for both conditions. Moreover, the pre-kainate data demonstrated a significant increase in high frequency bands in day 5 of examination, when compared with day 4. For high frequency bands post-kainate recordings, a significant increase was also shown between day 3 and day 4 of kainic acid administration (Figure 4, Table 2). This might suggest a plastic redistribution of the motor cortex activity as a reaction to the repeated kainic acid cerebellaras a reaction to the injections across days. The increase in gamma band in the pre-kainate state concomitant with the increase in the active wake behavior and decrease in the average dystonia score in day 4 and 5, despite the enhanced EMG activation, suggests a possible adaptive process to correct the dystonic behavior.

#### Somatosensory Cortex ECoG Power Spectral Density

**Figure 5:**
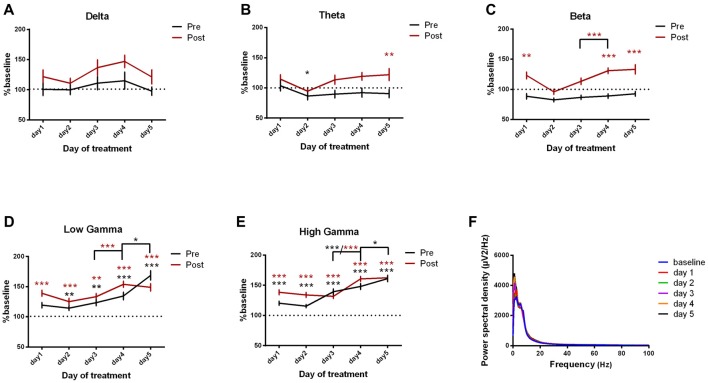
Power spectral density of somatosensory cortex ECoGs represented as percentage from baseline day across different bands. **(A)** Delta (0.5–3.5 Hz). **(B)** Theta (4–12 Hz). **(C)** Beta (13–30 Hz). **(D)** Low-gamma (30.5–48 Hz). **(E)** High-gamma (52–100 Hz) oscillations from Baseline day to day 1, day 2, day 3, day 4, day 5 of intracerebellar kainic acid administration. **(F)** Average ECoG power spectral density evolution from baseline day to the 5th day. For each frequency band, the spectra are expressed as average ± the SEM (*p < 0.05, **p < 0.01, ***p < 0.001, repeated measures ANOVA, Dunnett’s multiple comparisons test, each day vs. baseline day with the values of baseline group being normalized as 100%, Table 3).

We next investigated the ECoG power spectral density for the somatosensory cortex and we found important progressive increases in high frequency bands, reaching the maximum in the last day of experiment. These results have been observed since the first day of recordings for both pre and post-administration periods (Figures 5A–F, Table 3). In addition, few significant changes were also found in the low-frequency bands (Figures 5A,B).

**Table 3:** Somatosensory cortex power spectral density bands.

Somatosensory cortex spectral density bands (Figure 5)	Test	*F*_(DFn,DFd)_	*P* value
Delta pre	1-way ANOVA	*F*_(5,610)_ = 0.7700	*P* = 0.5716
day0 vs. day1	+Dunnett’s multiple comparisons test		ns
day0 vs. day2	+Dunnett’s multiple comparisons test		ns
day0 vs. day3	+Dunnett’s multiple comparisons test		ns
day0 vs. day4	+Dunnett’s multiple comparisons test		ns
day0 vs. day5	+Dunnett’s multiple comparisons test		ns
Delta post	1-way ANOVA	*F*_(5,603)_ = 1.380	*P* = 0.2301
day0 vs. day1	+Dunnett’s multiple comparisons test		ns
day0 vs. day2	+Dunnett’s multiple comparisons test		ns
day0 vs. day3	+Dunnett’s multiple comparisons test		ns
day0 vs. day4	+Dunnett’s multiple comparisons test		ns
day0 vs. day5	+Dunnett’s multiple comparisons test		ns
Theta pre	1-way ANOVA	*F*_(5,1507)_ = 1.827	*P* = 0.1045
day0 vs. day1	+Dunnett’s multiple comparisons test		ns
day0 vs. day2	+Dunnett’s multiple comparisons test		*
day0 vs. day3	+Dunnett’s multiple comparisons test		ns
day0 vs. day4	+Dunnett’s multiple comparisons test		ns
day0 vs. day5	+Dunnett’s multiple comparisons test		ns
Theta post	1-way ANOVA	*F*_(5,1473)_ = 3.733	*P* = 0.0023
day0 vs. day1	+Dunnett’s multiple comparisons test		ns
day0 vs. day2	+Dunnett’s multiple comparisons test		ns
day0 vs. day3	+Dunnett’s multiple comparisons test		ns
day0 vs. day4	+Dunnett’s multiple comparisons test		ns
day0 vs. day5	+Dunnett’s multiple comparisons test		**
Beta pre	1-way ANOVA	*F*_(5,3146)_ = 1.715	*P* = 0.1277
day0 vs. day1	+Dunnett’s multiple comparisons test		ns
day0 vs. day2	+Dunnett’s multiple comparisons test		ns
day0 vs. day3	+Dunnett’s multiple comparisons test		ns
day0 vs. day4	+Dunnett’s multiple comparisons test		ns
day0 vs. day5	+Dunnett’s multiple comparisons test		ns
Beta post	1-way ANOVA	*F*_(5,3039)_ = 14.59	*P* < 0.0001
day0 vs. day1	+Dunnett’s multiple comparisons test		**
day0 vs. day2	+Dunnett’s multiple comparisons test		ns
day0 vs. day3	+Dunnett’s multiple comparisons test		ns
day0 vs. day4	+Dunnett’s multiple comparisons test		***
day0 vs. day5	+Dunnett’s multiple comparisons test		***
Low gamma pre	1-way ANOVA	*F*_(5,3162)_ = 23.94	*P* < 0.0001
day0 vs. day1	+Dunnett’s multiple comparisons test		ns
day0 vs. day2	+Dunnett’s multiple comparisons test		**
day0 vs. day3	+Dunnett’s multiple comparisons test		**
day0 vs. day4	+Dunnett’s multiple comparisons test		***
day0 vs. day5	+Dunnett’s multiple comparisons test		***
Low gamma post	1-way ANOVA	*F*_(5,3126)_ = 17.70	*P* < 0.0001
day0 vs. day1	+Dunnett’s multiple comparisons test		***
day0 vs. day2	+Dunnett’s multiple comparisons test		***
day0 vs. day3	+Dunnett’s multiple comparisons test		**
day0 vs. day4	+Dunnett’s multiple comparisons test		***
day0 vs. day5	+Dunnett’s multiple comparisons test		***
High gamma pre	1-way ANOVA	*F*_(5,8530)_ = 85.14	*P* < 0.0001
day0 vs. day1	+Dunnett’s multiple comparisons test		***
day0 vs. day2	+Dunnett’s multiple comparisons test		***
day0 vs. day3	+Dunnett’s multiple comparisons test		***
day0 vs. day4	+Dunnett’s multiple comparisons test		***
day0 vs. day5	+Dunnett’s multiple comparisons test		***
High gamma post	1-way ANOVA	*F*_(5,8439)_ = 64.12	*P* < 0.0001
day0 vs. day1	+Dunnett’s multiple comparisons test		***
day0 vs. day2	+Dunnett’s multiple comparisons test		***
day0 vs. day3	+Dunnett’s multiple comparisons test		***
day0 vs. day4	+Dunnett’s multiple comparisons test		***
day0 vs. day5	+Dunnett’s multiple comparisons test		***
Delta pre			
day3 vs. day4	Mann Whitney test		*P* = 0.6585
day4 vs. day5	Mann Whitney test		*P* = 0.9723
Delta post			
day3 vs. day4	Mann Whitney test		*P* = 0.5757
day4 vs. day5	Mann Whitney test		*P* = 0.1021
Theta pre			
day3 vs. day4	Mann Whitney test		*P* = 0.5030
day4 vs. day5	Mann Whitney test		*P* = 0.6922
Theta post			
day3 vs. day4	Mann Whitney test		*P* = 0.0551
day4 vs. day5	Mann Whitney test		*P* = 0.8825
Beta pre			
day3 vs. day4	Mann Whitney test		*P* = 0.3119
day4 vs. day5	Mann Whitney test		*P* = 0.6042
Beta post			
day3 vs. day4	Mann Whitney test		*P* < 0.0001
day4 vs. day5	Mann Whitney test		*P* = 0.6332
Low gamma pre			
day3 vs. day4	Mann Whitney test		*P* = 0.1936
day4 vs. day5	Mann Whitney test		*P* = 0.0122
Low gamma post			
day3 vs. day4	Mann Whitney test		*P* < 0.0001
day4 vs. day5	Mann Whitney test		*P* = 0.5692
High gamma pre			
day3 vs. day4	Mann Whitney test		*P* < 0.0001
day4 vs. day5	Mann Whitney test		*P* = 0.0125
High gamma post			
day3 vs. day4	Mann Whitney test		*P* < 0.0001
day4 vs. day5	Mann Whitney test		*P* = 0.0806

Statistical data. *p < 0.05, **p < 0.01, ***p < 0.001, ns, not significant.

Furthermore, for post-kainate recordings, we noticed a significant increase in the somatosensory ECoG power spectral density for all high-frequency bands in day 4 of dystonia, when compared to day 3. Also, the pre-kainate data revealed an important increase in gamma band in the 4th and 5th day, when compared to the previous one (Figures 5C–E). Our results show that increases occur especially in the last 2 days of examination for both pre-kainate and post-kainate recordings.

#### Parietal Cortex ECoG Power Spectral Density

**Figure 6:**
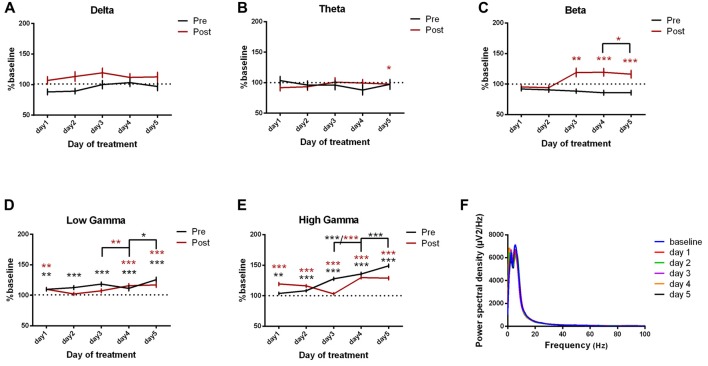
Power spectral density of parietal cortex ECoGs represented as percentage from baseline day across different bands. **(A)** Delta (0.5–3.5 Hz). **(B)** Theta (4–12 Hz). **(C)** Beta (13–30 Hz). **(D)** Low-gamma (30.5–48 Hz). **(E)** High-gamma (52–100 Hz) oscillations from baseline day to day 1, day 2, day 3, day 4, day 5 of intracerebellar kainic acid administration. **(F)** Average ECoG power spectral density evolution from baseline day to the 5th day. For each frequency band, the spectra are expressed as average ± the SEM (*p < 0.05, **p < 0.01, ***p < 0.001, repeated measures ANOVA, Dunnett’s multiple comparisons test, each day vs. baseline day with the values of baseline group being normalized as 100%, Table 4).

In addition, we investigated the changes in the parietal cortex ECoGs (Figures 6A–F, Table 4) and we found an important increase in power spectral density before and after the kainic acid administration in the high frequency bands (Figures 6C–E). In gamma band, this increase was observed across all days of experiment. The parietal cortex revealed higher power spectral densities for pre-kainate recordings in high-gamma band than for post-kainate ones. For the low-frequency bands, only theta showed a significant decrease in day 5 of post-kainate recordings (Figures 6A,B).

**Table 4:** Parietal cortex power spectral density bands.

Parietal cortex spectral density bands (Figure 6)	Test	*F*_(DFn,DFd)_	*P* value
Delta pre	1-way ANOVA	*F*_(5,624)_ = 1.561	*P* = 0.1692
day0 vs. day1	+Dunnett’s multiple comparisons test		ns
day0 vs. day2	+Dunnett’s multiple comparisons test		ns
day0 vs. day3	+Dunnett’s multiple comparisons test		ns
day0 vs. day4	+Dunnett’s multiple comparisons test		ns
day0 vs. day5	+Dunnett’s multiple comparisons test		ns
Delta post	1-way ANOVA	*F*_(5,624)_ = 1.457	*P* = 0.2021
day0 vs. day1	+Dunnett’s multiple comparisons test		ns
day0 vs. day2	+Dunnett’s multiple comparisons test		ns
day0 vs. day3	+Dunnett’s multiple comparisons test		ns
day0 vs. day4	+Dunnett’s multiple comparisons test		ns
day0 vs. day5	+Dunnett’s multiple comparisons test		ns
Theta pre	1-way ANOVA	*F*_(5,1524)_ = 0.4282	*P* = 0.8292
day0 vs. day1	+Dunnett’s multiple comparisons test		ns
day0 vs. day2	+Dunnett’s multiple comparisons test		ns
day0 vs. day3	+Dunnett’s multiple comparisons test		ns
day0 vs. day4	+Dunnett’s multiple comparisons test		ns
day0 vs. day5	+Dunnett’s multiple comparisons test		ns
Theta post	1-way ANOVA	*F*_(5,1524)_ = 2.904	*P* = 0.0129
day0 vs. day1	+Dunnett’s multiple comparisons test		ns
day0 vs. day2	+Dunnett’s multiple comparisons test		ns
day0 vs. day3	+Dunnett’s multiple comparisons test		ns
day0 vs. day4	+Dunnett’s multiple comparisons test		ns
day0 vs. day5	+Dunnett’s multiple comparisons test		*
Beta pre	1-way ANOVA	*F*_(5,3216)_ = 0.6552	*P* = 0.6575
day0 vs. day1	+Dunnett’s multiple comparisons test		ns
day0 vs. day2	+Dunnett’s multiple comparisons test		ns
day0 vs. day3	+Dunnett’s multiple comparisons test		ns
day0 vs. day4	+Dunnett’s multiple comparisons test		ns
day0 vs. day5	+Dunnett’s multiple comparisons test		ns
Beta post	1-way ANOVA	*F*_(5,3144)_ = 14.86	*P* < 0.0001
day0 vs. day1	+Dunnett’s multiple comparisons test		ns
day0 vs. day2	+Dunnett’s multiple comparisons test		ns
day0 vs. day3	+Dunnett’s multiple comparisons test		**
day0 vs. day4	+Dunnett’s multiple comparisons test		***
day0 vs. day5	+Dunnett’s multiple comparisons test		***
Low gamma pre	1-way ANOVA	*F*_(5,3234)_ = 15.84	*P* < 0.0001
day0 vs. day1	+Dunnett’s multiple comparisons test		ns
day0 vs. day2	+Dunnett’s multiple comparisons test		**
day0 vs. day3	+Dunnett’s multiple comparisons test		***
day0 vs. day4	+Dunnett’s multiple comparisons test		***
day0 vs. day5	+Dunnett’s multiple comparisons test		***
Low gamma post	1-way ANOVA	*F*_(5,3234)_ = 14.39	*P* < 0.0001
day0 vs. day1	+Dunnett’s multiple comparisons test		**
day0 vs. day2	+Dunnett’s multiple comparisons test		ns
day0 vs. day3	+Dunnett’s multiple comparisons test		ns
day0 vs. day4	+Dunnett’s multiple comparisons test		***
day0 vs. day5	+Dunnett’s multiple comparisons test		***
High gamma pre	1-way ANOVA	*F*_(5,8724)_ = 137.3	*P* < 0.0001
day0 vs. day1	+Dunn multiple comparisons test		**
day0 vs. day2	+Dunn multiple comparisons test		***
day0 vs. day3	+Dunn multiple comparisons test		***
day0 vs. day4	+Dunn multiple comparisons test		***
day0 vs. day5	+Dunn multiple comparisons test		***
High gamma post	1-way ANOVA	*F*_(5,8730)_ = 53.57	*P* < 0.0001
day0 vs. day1	+Dunn multiple comparisons test		***
day0 vs. day2	+Dunn multiple comparisons test		***
day0 vs. day3	+Dunn multiple comparisons test		***
day0 vs. day4	+Dunn multiple comparisons test		***
day0 vs. day5	+Dunn multiple comparisons test		***
Delta pre			
day3 vs. day4	Mann Whitney test		*P* = 0.5624
day4 vs. day5	Mann Whitney test		*P* = 0.3381
Delta post			
day3 vs. day4	Mann Whitney test		*P* = 0.6417
day4 vs. day5	Mann Whitney test		*P* = 0.7438
Theta pre			
day3 vs. day4	Mann Whitney test		*P* = 0.9601
day4 vs. day5	Mann Whitney test		*P* = 0.7127
Theta post			
day3 vs. day4	Mann Whitney test		*P* = 0.1778
day4 vs. day5	Mann Whitney test		*P* = 0.1067
Beta pre			
day3 vs. day4	Mann Whitney test		*P* = 0.5185
day4 vs. day5	Mann Whitney test		*P* = 0.8966
Beta post			
day3 vs. day4	Mann Whitney test		*P* = 0.1108
day4 vs. day5	Mann Whitney test		*P* = 0.0165
Low gamma pre			
day3 vs. day4	Mann Whitney test		*P* = 0.9334
day4 vs. day5	Mann Whitney test		*P* = 0.0106
Low gamma post			
day3 vs. day4	Mann Whitney test		*P* = 0.0049
day4 vs. day5	Mann Whitney test		*P* = 0.0821
High gamma pre			
day3 vs. day4	Mann Whitney test		*P* < 0.0001
day4 vs. day5	Mann Whitney test		*P* < 0.0001
High gamma post			
day3 vs. day4	Mann Whitney test		*P* < 0.0001
day4 vs. day5	Mann Whitney test		*P* = 0.7735

Statistical data. *p < 0.05, **p < 0.01, ***p < 0.001, ns, not significant.

Interestingly, when compared to the previous day, the parietal cortex power spectral density also increased significantly in the last 2 days of examination in the high-frequency bands, for both pre and post-kainate recordings (Figures 6C–E).

#### Motor-Ipsilateral Somatosensory Cortices Coherence

**Figure 7:**
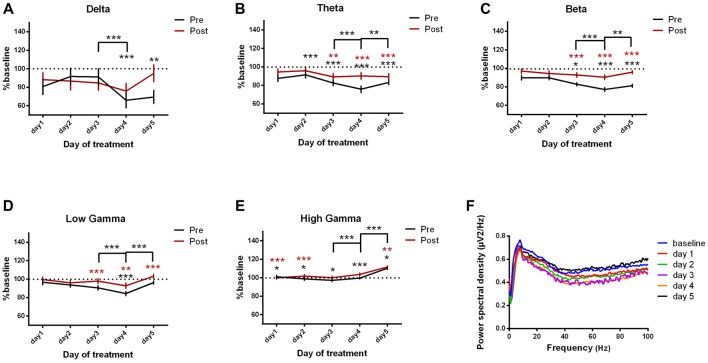
Cortical coherence between motor cortex and somatosensory cortices ECoGs (represented as percentage from baseline day) across different bands. **(A)** Delta (0.5–3.5 Hz). **(B)** Theta (4–12 Hz). **(C)** Beta (13–30 Hz). **(D)** Low-gamma (30.5–48 Hz). **(E)** High-gamma (52– 100 Hz) oscillations from baseline day to day 1, day 2, day 3, day 4, day 5 of intracerebellar kainic acid administration. **(F)** Average ECoG power spectral density evolution from baseline day to the 5th day. For each frequency band, the spectra are expressed as average ± the SEM (*p < 0.05, **p < 0.01, ***p < 0.001, repeated measures ANOVA, Dunnett’s multiple comparisons test, each day vs. baseline day with the values of baseline group being normalized as 100%, Table 5).

We then assessed the coherence between motor and ipsilateral somatosensory cortices (Figures 7A–F, Table 5). Notably, when comparing it to the baseline day, the cortical coherence of motor-somatosensory post-kainate recordings was significantly reduced in theta and beta bands (Figures 7B,C). Conversely, high gamma band showed increased coherence since the 1st day of kainate administration, with a recorded maximum in day 5 (Figure 7E). Pre-kainate administration periods revealed significantly lower motor-somatosensory coherence in low-frequency bands (Figures 7A,B). In the high frequency bands, except for day 5 in high gamma band, the pre-kainate coherence was also decreased (Figures 7C–E).

**Table 5:** Motor-ipsilateral somatosensory coherence.

Motor-somatosensory coherence (Figure 7)	Test	*F*_(DFn,DFd)_	*P* value
Delta pre	1-way ANOVA	*F*_(5,666)_ = 4.469	*P* = 0.0005
day0 vs. day1	+Dunnett’s multiple comparisons test		ns
day0 vs. day2	+Dunnett’s multiple comparisons test		ns
day0 vs. day3	+Dunnett’s multiple comparisons test		ns
day0 vs. day4	+Dunnett’s multiple comparisons test		***
day0 vs. day5	+Dunnett’s multiple comparisons test		**
Delta post	1-way ANOVA	*F*_(5,631)_ = 2.443	*P* = 0.0331
day0 vs. day1	+Dunnett’s multiple comparisons test		ns
day0 vs. day2	+Dunnett’s multiple comparisons test		ns
day0 vs. day3	+Dunnett’s multiple comparisons test		ns
day0 vs. day4	+Dunnett’s multiple comparisons test		ns
day0 vs. day5	+Dunnett’s multiple comparisons test		ns
Theta pre	1-way ANOVA	*F*_(5,1626)_ = 14.69	*P* < 0.0001
day0 vs. day1	+Dunnett’s multiple comparisons test		ns
day0 vs. day2	+Dunnett’s multiple comparisons test		***
day0 vs. day3	+Dunnett’s multiple comparisons test		***
day0 vs. day4	+Dunnett’s multiple comparisons test		***
day0 vs. day5	+Dunnett’s multiple comparisons test		***
Theta post	1-way ANOVA	*F*_(5,1541)_ = 11.81	*P* < 0.0001
day0 vs. day1	+Dunnett’s multiple comparisons test		ns
day0 vs. day2	+Dunnett’s multiple comparisons test		ns
day0 vs. day3	+Dunnett’s multiple comparisons test		**
day0 vs. day4	+Dunnett’s multiple comparisons test		***
day0 vs. day5	+Dunnett’s multiple comparisons test		***
Beta pre	1-way ANOVA	*F*_(5,3426)_ = 14.07	*P* < 0.0001
day0 vs. day1	+Dunnett’s multiple comparisons test		ns
day0 vs. day2	+Dunnett’s multiple comparisons test		ns
day0 vs. day3	+Dunnett’s multiple comparisons test		*
day0 vs. day4	+Dunnett’s multiple comparisons test		***
day0 vs. day5	+Dunnett’s multiple comparisons test		***
Beta post	1-way ANOVA	*F*_(5,3179)_ = 16.68	*P* < 0.0001
day0 vs. day1	+Dunnett’s multiple comparisons test		ns
day0 vs. day2	+Dunnett’s multiple comparisons test		ns
day0 vs. day3	+Dunnett’s multiple comparisons test		***
day0 vs. day4	+Dunnett’s multiple comparisons test		***
day0 vs. day5	+Dunnett’s multiple comparisons test		***
Low gamma pre	1-way ANOVA	*F*_(5,3450)_ = 9.262	*P* < 0.0001
day0 vs. day1	+Dunnett’s multiple comparisons test		ns
day0 vs. day2	+Dunnett’s multiple comparisons test		ns
day0 vs. day3	+Dunnett’s multiple comparisons test		ns
day0 vs. day4	+Dunnett’s multiple comparisons test		***
day0 vs. day5	+Dunnett’s multiple comparisons test		ns
Low gamma post	1-way ANOVA	*F*_(5,3270)_ = 15.02	*P* < 0.0001
day0 vs. day1	+Dunnett’s multiple comparisons test		ns
day0 vs. day2	+Dunnett’s multiple comparisons test		ns
day0 vs. day3	+Dunnett’s multiple comparisons test		***
day0 vs. day4	+Dunnett’s multiple comparisons test		**
day0 vs. day5	+Dunnett’s multiple comparisons test		***
High gamma pre	1-way ANOVA	*F*_(5,9306)_ = 12.67	*P* < 0.0001
day0 vs. day1	+Dunnett’s multiple comparisons test		*
day0 vs. day2	+Dunnett’s multiple comparisons test		*
day0 vs. day3	+Dunnett’s multiple comparisons test		*
day0 vs. day4	+Dunnett’s multiple comparisons test		***
day0 vs. day5	+Dunnett’s multiple comparisons test		*
High gamma post	1-way ANOVA	*F*_(5,8827)_ = 32.84	*P* < 0.0001
day0 vs. day1	+Dunnett’s multiple comparisons test		***
day0 vs. day2	+Dunnett’s multiple comparisons test		***
day0 vs. day3	+Dunnett’s multiple comparisons test		ns
day0 vs. day4	+Dunnett’s multiple comparisons test		ns
day0 vs. day5	+Dunnett’s multiple comparisons test		**
Delta pre			
day3 vs. day4	Mann Whitney test		*P* = 0.0005
day4 vs. day5	Mann Whitney test		*P* = 0.0790
Delta post			
day3 vs. day4	Mann Whitney test		*P* = 0.4940
day4 vs. day5	Mann Whitney test		*P* = 0.8762
Theta pre			
day3 vs. day4	Mann Whitney test		*P* < 0.0001
day4 vs. day5	Mann Whitney test		*P* = 0.0077
Theta post			
day3 vs. day4	Mann Whitney test		*P* = 0.9324
day4 vs. day5	Mann Whitney test		*P* = 0.1112
Beta pre			
day3 vs. day4	Mann Whitney test		*P* < 0.0001
day4 vs. day5	Mann Whitney test		*P* = 0.0063
Beta post			
day3 vs. day4	Mann Whitney test		*P* = 0.3153
day4 vs. day5	Mann Whitney test		*P* = 0.5027
Low gamma pre			
day3 vs. day4	Mann Whitney test		*P* < 0.0001
day4 vs. day5	Mann Whitney test		*P* < 0.0001
Low gamma post			
day3 vs. day4	Mann Whitney test		*P* = 0.1747
day4 vs. day5	Mann Whitney test		*P* = 0.5429
High gamma pre			
day3 vs. day4	Mann Whitney test		*P* < 0.0001
day4 vs. day5	Mann Whitney test		*P* < 0.0001
High gamma post			
day3 vs. day4	Mann Whitney test		*P* = 0.8062
day4 vs. day5	Mann Whitney test		*P* = 0.2107

Statistical data. *p < 0.05, **p < 0.01, ***p < 0.001, ns, not significant.

We further investigated the changes that occurred in the last 2 days of experiment. For pre-kainate recordings, the coherence decreased in day 4 (when comparing it to day 3) in all frequency bands, except high gamma (Figures 7A–E). Starting with day 4, an increasing trend has been observed for all frequency bands. Day 5 revealed significantly higher coherences in theta and high frequency bands (Figures 7B–E). In conclusion, motor-somatosensory coherence decreased in all frequency bands for both pre and post-kainate periods, but, since day 4, it started to increase.

#### Motor-Ipsilateral Parietal Cortices Coherence

**Figure 8:**
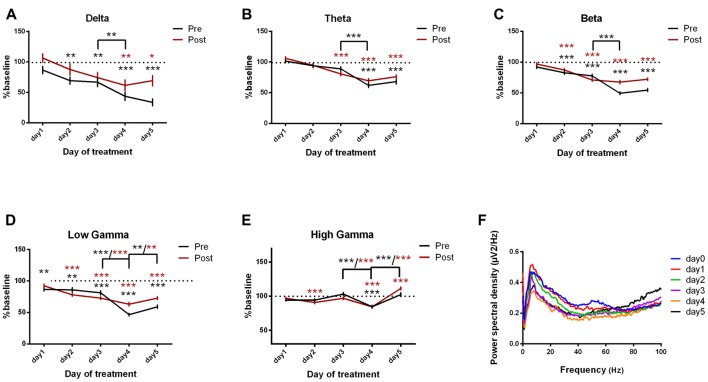
Cortical coherence between motor cortex and parietal cortices ECoGs (represented as percentage from baseline day) across different bands. **(A)** Delta (0.5–3.5 Hz). **(B)** Theta (4–12 Hz). **(C)** Beta (13–30 Hz). **(D)** Low-gamma (30.5–48 Hz). **(E)** High-gamma (52–100 Hz) oscillations from baseline day to day 1, day 2, day 3, day 4, day 5 of intracerebellar kainic acid administration. **(F)** Average ECoG power spectral density evolution from baseline day to the 5th day in post-kainate state. For each frequency band, the spectra are expressed as average ± the SEM (*p < 0.05, **p < 0.01, ***p < 0.001, repeated measures ANOVA, Dunnett’s multiple comparisons test, each day vs. baseline day with the values of baseline group being normalized as 100%, Table 6).

Motor-parietal cortex coherence was significantly reduced from the baseline day to all dystonia days across all the tested frequency bands (Figures 8A–F, Table 6). For the post-kainate recordings, we found an increased coherence in high gamma band in day 5 of dystonia.

**Table 6:** Motor-ipsilateral parietal coherence.

Motor-parietal coherence (Figure 8)	Test	*F*_(DFn,DFd)_	*P* value
Delta pre	1-way ANOVA	*F*_(5,351)_ = 14.51	*P* < 0.0001
day0 vs. day1	+Dunnett’s multiple comparisons test		ns
day0 vs. day2	+Dunnett’s multiple comparisons test		**
day0 vs. day3	+Dunnett’s multiple comparisons test		**
day0 vs. day4	+Dunnett’s multiple comparisons test		***
day0 vs. day5	+Dunnett’s multiple comparisons test		***
Delta post	1-way ANOVA	*F*_(5,365)_ = 4.809	*P* = 0.0003
day0 vs. day1	+Dunnett’s multiple comparisons test		ns
day0 vs. day2	+Dunnett’s multiple comparisons test		ns
day0 vs. day3	+Dunnett’s multiple comparisons test		ns
day0 vs. day4	+Dunnett’s multiple comparisons test		**
day0 vs. day5	+Dunnett’s multiple comparisons test		*
Theta pre	1-way ANOVA	*F*_(5,861)_ = 26.64	*P* < 0.0001
day0 vs. day1	+Dunnett’s multiple comparisons test		ns
day0 vs. day2	+Dunnett’s multiple comparisons test		ns
day0 vs. day3	+Dunnett’s multiple comparisons test		ns
day0 vs. day4	+Dunnett’s multiple comparisons test		***
day0 vs. day5	+Dunnett’s multiple comparisons test		***
Theta post	1-way ANOVA	*F*_(5,895)_ = 22.43	*P* < 0.0001
day0 vs. day1	+Dunnett’s multiple comparisons test		ns
day0 vs. day2	+Dunnett’s multiple comparisons test		ns
day0 vs. day3	+Dunnett’s multiple comparisons test		***
day0 vs. day4	+Dunnett’s multiple comparisons test		***
day0 vs. day5	+Dunnett’s multiple comparisons test		***
Beta pre	1-way ANOVA	*F*_(5,1779)_ = 64.37	*P* < 0.0001
day0 vs. day1	+Dunnett’s multiple comparisons test		ns
day0 vs. day2	+Dunnett’s multiple comparisons test		***
day0 vs. day3	+Dunnett’s multiple comparisons test		***
day0 vs. day4	+Dunnett’s multiple comparisons test		***
day0 vs. day5	+Dunnett’s multiple comparisons test		***
Beta post	1-way ANOVA	*F*_(5,1849)_ = 33.06	*P* < 0.0001
day0 vs. day1	+Dunnett’s multiple comparisons test		ns
day0 vs. day2	+Dunnett’s multiple comparisons test		***
day0 vs. day3	+Dunnett’s multiple comparisons test		***
day0 vs. day4	+Dunnett’s multiple comparisons test		***
day0 vs. day5	+Dunnett’s multiple comparisons test		***
Low gamma pre	1-way ANOVA	*F*_(5,1830)_ = 46.99	*P* < 0.0001
day0 vs. day1	+Dunnett’s multiple comparisons test		**
day0 vs. day2	+Dunnett’s multiple comparisons test		**
day0 vs. day3	+Dunnett’s multiple comparisons test		***
day0 vs. day4	+Dunnett’s multiple comparisons test		***
day0 vs. day5	+Dunnett’s multiple comparisons test		***
Low gamma post	1-way ANOVA	*F*_(5,1902)_ = 29.69	*P* < 0.0001
day0 vs. day1	+Dunnett’s multiple comparisons test		ns
day0 vs. day2	+Dunnett’s multiple comparisons test		***
day0 vs. day3	+Dunnett’s multiple comparisons test		***
day0 vs. day4	+Dunnett’s multiple comparisons test		***
day0 vs. day5	+Dunnett’s multiple comparisons test		***
High gamma pre	1-way ANOVA	*F*_(5,4941)_ = 11.30	*P* < 0.0001
day0 vs. day1	+Dunnett’s multiple comparisons test		ns
day0 vs. day2	+Dunnett’s multiple comparisons test		ns
day0 vs. day3	+Dunnett’s multiple comparisons test		ns
day0 vs. day4	+Dunnett’s multiple comparisons test		***
day0 vs. day5	+Dunnett’s multiple comparisons test		ns
High gamma post	1-way ANOVA	*F*_(5,5135)_ = 26.34	*P* < 0.0001
day0 vs. day1	+Dunnett’s multiple comparisons test		ns
day0 vs. day2	+Dunnett’s multiple comparisons test		***
day0 vs. day3	+Dunnett’s multiple comparisons test		ns
day0 vs. day4	+Dunnett’s multiple comparisons test		***
day0 vs. day5	+Dunnett’s multiple comparisons test		***
Delta pre			
day3 vs. day4	Mann Whitney test		*P* = 0.0078
day4 vs. day5	Mann Whitney test		*P* = 0.2144
Delta post			
day3 vs. day4	Mann Whitney test		*P* = 0.1434
day4 vs. day5	Mann Whitney test		*P* = 0.4075
Theta pre			
day3 vs. day4	Mann Whitney test		*P* < 0.0001
day4 vs. day5	Mann Whitney test		*P* = 0.2441
Theta post			
day3 vs. day4	Mann Whitney test		*P* = 0.0502
day4 vs. day5	Mann Whitney test		*P* = 0.1605
Beta pre			
day3 vs. day4	Mann Whitney test		*P* < 0.0001
day4 vs. day5	Mann Whitney test		*P* = 0.2699
Beta post			
day3 vs. day4	Mann Whitney test		*P* = 0.7344
day4 vs. day5	Mann Whitney test		*P* = 0.3052
Low gamma pre			
day3 vs. day4	Mann Whitney test		*P* < 0.0001
day4 vs. day5	Mann Whitney test		*P* = 0.0017
Low gamma post			
day3 vs. day4	Mann Whitney test		*P* = 0.0003
day4 vs. day5	Mann Whitney test		*P* = 0.0015
High gamma pre			
day3 vs. day4	Mann Whitney test		*P* = 0.0001
day4 vs. day5	Mann Whitney test		*P* < 0.0001
High gamma post			
day3 vs. day4	Mann Whitney test		*P* < 0.0001
day4 vs. day5	Mann Whitney test		*P* < 0.0001

Statistical data. *p < 0.05, **p < 0.01, ***p < 0.001, ns, not significant.

Over the last 2 days of recordings we found that, between day 4 and day 5, the motor-parietal cortex coherence was significantly increased in high-frequency bands for the pre and post-administration periods. However, between day 3 and day 4, a significant decrease was observed for all pre-kainate data and also for post-kainate recordings in the high frequency bands (Figures 8A–E).

The analysis of motor-somatosensory and motor-parietal coherences coupled with dystonia scores suggest a negative correlation between the average dystonia score and high gamma frequency band.

We also calculated the imaginary part of coherence (Nolte et al., 2004; Stam et al., 2007) to estimate the coherence avoiding contamination by volume conduction. We found a very clear imaginary coherence coupling in low frequencies and in gamma band (Supplementary Figures S1C,D) that exclude volume conduction. We also yielded consistent results of the quantification of coherences in pre and post kainate injections in the cerebellum compared with the real part of the coherence, with an increased coherence post kainate injection (Supplementary Figures S1A,B).

#### Changes in Neural Activity Predict Changes in Motor Behavior

We assessed the link between behavior, dystonia and neuronal activity by computing the correlations between dystonic behavior (dystonia score or active wake) and the ECoG power spectral densities for motor, somatosensory and parietal cortices and also for motor-somatosensory and motor-parietal coherences (Supplementary Tables S1–S10). We found correlations notably in the first day of dystonia (Supplementary Figures S2–S6). The following days there was little or no relationship between dystonia scores and motor, sensory or parietal cortices activity because of the lack of variability for dystonia scores (Supplementary Tables S1–S10). Indeed, we found a very severe phenotype of dystonia after kainate injections in all our mice (Figure 2), while we found a reorganization of cortical neuronal activity across motor, somatosensory and parietal network (Figures 4–8). Specifically, for the motor cortex, we found a negative linear regression between dystonia score and motor cortex power spectral density in high gamma band in day 1 and day 4 (Supplementary Figure S2A, Supplementary Table S2). Also, a positive correlation was found for delta band and AW% (post-administration) in day 4 (Supplementary Figure S2C, Supplementary Table S1). Somatosensory cortex power spectral density revealed negative linear regressions with dystonia score in beta (day 3), low gamma (day 3, day 4) and high gamma bands (day 4; Supplementary Figure S3A, Supplementary Table S4). Also, positive correlations were found with AW% in delta (pre-administration, in day 3 and day 5 and post-administration in day 4; Supplementary Figures S3B,C, Supplementary Table S3). In the parietal cortex, positive correlations were found between delta power spectral density and AW% (post-administration, day 1; Supplementary Figure S4C, Supplementary Table S5), as well as between delta and dystonia score in day 3 (Supplementary Figure S4A, Supplementary Table S6). We further examined the motor-somatosensory and motor-parietal cortices coherence in relationship with behavior. For the motor-somatosensory cortex we found a negative correlation with AW% (post-administration) in delta (day 1, day 2), theta (day 1, day 2), beta (day 2) and low gamma (day 2; Supplementary Figure S5C, Supplementary Table S7). The motor-parietal cortex coherence was positively correlated with dystonia score in delta (day 1; Supplementary Figure S6A, Supplementary Table S10).

## Discussion

Our study examined the relationship between the activation of the cerebellar cortex with kainate and the intra-cortical oscillatory activities in normal and dystonic conditions. We found functional reorganization of multiple cerebral cortical areas and new coordination of their activity. In this model (Pizoli et al., 2002), the dystonia occured only after the kainic acid administration and it lasted for 3 h, without visible signs of dystonia in the next day. The injection was performed for five consecutive days. We obtained a phenomenon of permanent adaptation with a change of baseline locomotor activity together with an ECoG high gamma band increase in the motor and parietal cortices. In addition, in the post-kainate state we noticed an adaptation in the motor circuit across days with an increase in muscular activity in day 4 and day 5, but with less signs of dystonia and with changes in power spectral of ECoG of all frequency bands in the motor circuit. Our results revealed less signs of dystonia in the last 2 days, coupled with a reduced motor-somatosensory coherence in all bands, except for day 5 in gamma band. The increase in the locomotor activity suggests improved control of muscular contraction across days of dystonia.

Furthermore, we found a reduction of the motor-somatosensory and motor-parietal cortex oscillations coherences in low and high frequency ranges, after cerebellar kainate injections. This data is consistent with other lines of recent evidence (Mantel et al., 2018) that writer’s cramp patients have reduced functional connectivity for the right motor and S1, supramarginal gyrus and also posterior parietal cortex. Interestingly, beginning day 4 of cerebellar kainate administration, intra-cortical coherence started to return to normal values. This phenomenon might be explained by a compensatory mechanism or a reduction of the sensibility of the kainate receptors after multiple administrations, even though the kainate might still have a muscular effect as indicated by the increase in mean frequency of EMG and median frequency of EMG consistent with a prolongation of muscle activation. This reflects the EMG pattern of movement in dystonia, characterized by excessive motor activation that can be due to excessive activation of antagonist muscles, redundant activation of muscles and maintenance of muscle activation producing abnormal muscle contraction and lack of coordination (Mima et al., 2000).

### Cerebellum and Dystonia

We found that mice developed sustained and reproducible dystonic motor behavior after daily kainate application on the cerebellar vermis in agreement with previous studies. Multiple applications of kainic acid into the cerebellar vermis resulted in increased neuronal activation (indicated by c-fos expression) in the cerebellum in all three layers of the cerebellar cortex and in the ventro-anterior thalamus. Also, Hsp70 expression was increased in the Purkinje cell layer and in the magnocellular part of the red nucleus, which is the first output of the cerebellar nuclei, suggesting that an altered neuronal network may be part of the pathogenesis of the disorder (Alvarez-Fischer et al., 2012). Moreover, kainic acid cerebellar application induced a significantly lower degree of dystonia in mice lacking Purkinje cells, this underlying the critical role of these cells in the pathogenesis of the disorder (Pizoli et al., 2002). Glutamate receptor activation, specifically AMPA receptor activation by kainic acid was necessary to produce dystonia, whereas a nonspecific increase in cerebellar excitability was not enough to induce dystonic behavior (Fan et al., 2012).

The cerebellum and the cerebello-forebrain pathways have been implicated in several animal models of dystonia (Chen et al., 2014; Shakkottai et al., 2017). Indeed, the cerebellar vermis was proven to be involved in controlling anticipatory postural motor behavior and to be connected to the motor cortex (Diedrichsen et al., 2005). It was previously believed that postural dystonia might be related to its impaired function (Coffman et al., 2011). Also, in human studies, cerebellar structural neuroimaging abnormalities such as atrophy (Delmaire et al., 2007), anatomical disturbance of cerebellar output (Niethammer et al., 2011) or lesions (Batla et al., 2015) have been reported. Additionally, PET imaging studies have revealed that many different forms of dystonia are characterized by abnormal increases in cerebellar metabolic activity (Hutchinson et al., 2000; Carbon et al., 2013). In mutant tottering mice, which exhibit paroxysmal dystonia due to an inherited defect affecting calcium channels, prior studies have shown that abnormal cerebellar output is essential for the generation of dystonic movements with slow oscillations occurring in the cerebellar cortex in relation with dystonic movements (Chen et al., 2009). In another genetic model of dystonia in the mice, a recent study showed that dystonia is eliminated following surgical removal of the cerebellum (Devanagondi et al., 2007). Cerebellectomy also relieved the motor symptoms in a genetic model of dystonia in the rat (LeDoux, 2011). In other genetic models of dystonia—*dt* rat, tottering mouse and mouse with invalidation of type 1 inositol triphosphate receptor in the cerebellum/brainstem—removal of the cerebellum, or only cerebellar Purkinje neurons or deep cerebellar nuclei, eliminates dystonic movements, showing that abnormalities in the cerebellum are the source of the movement disorders (LeDoux et al., 1993, 1995; Campbell et al., 1999; Neychev et al., 2008; Hisatsune et al., 2013). In addition, the block of the olivocerebellar excitatory neurotransmission has been shown to eliminate Purkinje cell complex spikes and to produce aberrant cerebellar nuclear activity while inducing dystonic behavior (White and Sillitoe, 2017). These findings lead to the proposal that dystonia in these models is linked to an increase in neuronal activity in the cerebellum. This hypothesis is confirmed by studies showing that excitation of the cerebellum by local application of the glutamate agonist kainic acid evokes in normal mice movements that have similarities with human dystonia (Pizoli et al., 2002). When dystonic movements were triggered by pharmacological stimulation of the cerebellum, microdialysis revealed significant reductions in striatal dopamine release (Pizoli et al., 2002). These results suggest that dystonia may occur from disruption of a motor network involving both the basal ganglia and the cerebellum (Chen et al., 2014; Bostan and Strick, 2018), rather than isolated dysfunction of only one motor system. By using conditional genetics to regionally limit cerebellar dysfunction, Raike et al. (2013) demonstrated that abnormalities restricted to cerebellar Purkinje cells are sufficient to cause dystonia and that the extent of cerebellar dysfunction determines the extent of dystonic movements. Recently it was shown that conditional knockout mice lacking type 1 inositol 1,4,5-trisphosphate receptor (IP3R1) specifically in the cerebellum and brainstem, experienced dystonia the symptoms of which were independent of the basal ganglia, and could be rescued by inactivation of the cerebellum, inferior olive or in the absence of Purkinje cells (Hisatsune et al., 2013). Heterozygous animals carrying the DYT1 dystonia mutation in the TOR1a gene exhibit no behavioral defect, but defects in the cerebello-cortical pathway are similar to those found in human non-manifesting gene carriers, confirming the role of this pathway in the penetrance of the disease (Uluğ et al., 2011). Therefore, the changes that we found in intra-cerebral oscillatory activity could be in part results from depressed cerebello-cortical coupling.

### Cerebellum, Oscillations and Dystonia

We found changes in the coordination of cortical networks following cerebellar kainate injections. Previous studies on dystonia examined cortical oscillations during simple movements without providing cerebellar activity. Dystonic patients have impaired movement-related beta decrease during simple movements in primary somatosensory cortices (Jin et al., 2011). Recently in focal hand dystonia patients, a significant decrease in high gamma power in the somatosensory cortex was identified during the preparation of simple movements of the affected hand compared to healthy subjects (Hinkley et al., 2012). Oscillation studies in deep structures were limited to the internal globus pallidus (Neumann et al., 2017), where a high gamma synchrony was observed when patients performed a reaction-time task with their unaffected hand. It was related to the scaling of ongoing movements (Brücke et al., 2012). Moreover, when performing voluntary movements, patients with primary dystonia showed an increase in synchronization in the high frequency range (Liu et al., 2008). It has also been suggested that, due to their neuronal activity synchronization in high frequency bands, the basal ganglia may contribute to hyperkinesias (Chen et al., 2006). These results suggest complex changes in oscillatory dynamics of high rhythms in dystonia.

We found changes in intra-cortical oscillations particularly in gamma band in motor, somatosensory and parietal cortices. Among the oscillations generated during intense neuronal communication, gamma rhythms appear to function as a temporal code, facilitating the dynamic formation of neuronal assemblies by permitting synchronous firing among multiple, spatially separate subpopulations of neurons (Schoffelen et al., 2005). The networks supporting gamma oscillations critically depend on the inhibitory neurotransmitter γ-aminobutyric acid (GABA; Cardin et al., 2009; Gallea et al., 2018). Gamma oscillations represent reference signals for polysensory integration (Mishra et al., 2007), sensory-motor coordination (Schoffelen et al., 2005) and formation of long-term memories through spike timing-dependent plasticity (STDP; Wespatat et al., 2004). The ability of the cerebellar cortex to generate rhythms within the gamma bands (30–80 and 80–160 Hz), as does the motor cortex, suggests that these rhythms may represent a common spatiotemporal code for the cortico-cerebellar dialog (Middleton et al., 2008). In humans, high gamma synchronization was observed in the cerebellum and the inferior parietal cortex during internal generation of decision and actions (Guggisberg et al., 2008) and in bilateral cerebellum after learning a bimanual complex motor tasks (Houweling et al., 2008). High gamma activity in the cerebellum and somatosensory cortex was observed during paced finger movement (Dalal et al., 2008). Our results of changes in coordination of neuronal activity across the cortical somatosensory/parietal network may underlie deficits in motor skills in dystonia.

### Compensatory Activity and Motor Circuit Plasticity

We found that the severity of dystonia was sustained during the first 3 days of cerebellar kainate administrations and it decreased for the last 2 days. This was coupled with increased activity particularly in gamma bands in motor cortex. This change might induce frequency modulations in cortical networks and basal ganglia and compensatory activities that can rescue the behavior. In our model, dystonia resulted from abnormalities in cerebellar cortical activity due to kainate administration and this induced subsequent compensatory activities in motor systems (Shakkottai et al., 2017). However, the role of the motor cortex in cerebellar-induced dystonia is limited. Indeed, cortical activity contributes to the severity of cerebellar-induced dystonic postured, but dystonia can manifest in the absence of overt cortical activity (Calderon et al., 2011). We also found a correlation between the behavior, dystonia and motor cortex activity notably during the first day of induction of dystonia suggesting that changes in motor cortex activity induced by changes in cerebellum could predict changes in motor behavior. However, in the following days there was little or no relationship between dystonia scores and motor cortex activity and compensatory or adaptive cortical activities were observed after multiple injections of kainate in cerebellum.

Gallea et al. (2018) suggested that there is a loss of cerebellar modulation of M1 in dystonia and that this originated from GABAergic changes in cerebellar structures that could be compensatory or adaptive.

We also observed changes in parietal cortex activities during dystonia. This result is in line with the abnormal activation of the parietal cortex that was found in dystonic patients without task-specific symptoms (Delnooz et al., 2013), as well as in task specific dystonia during non-symptomatic tasks or at rest (Gallea et al., 2016). An abnormal processing of multisensory input is a key pathophysiologic concept in dystonia because sensory activation can improve dystonic symptoms (Stamelou et al., 2012), and amelioration of reduced parietal activity has been shown during sensory activation in cervical dystonia (Mantel et al., 2018).

While human studies have established a cerebellar contribution to cortical plasticity (Popa et al., 2013, 2018), the counterpart phenomenon has not yet been studied in animal models. Indirect electron-microscopy evidence the presence of plastic changes in the cerebello-thalamic pathway (Aumann and Horne, 1999). The cerebello-thalamo-cortical pathway contacts the pyramidal neurons via a di- or trisynaptic pathway, and notably excites the pyramidal tract neurons (Futami et al., 1986) and the cortico-thalamic neurons (Na et al., 1997). The cerebellum indeed regulates motor cortex excitability (Oulad Ben Taib et al., 2005). Moreover, we recently demonstrated an important role of the cerebellum in the somatosensory coupling of the cortex where it was found that the cerebellum baselines the gamma-band synchronization of the sensori-motor cortex during active tactile exploration in the rodent (Popa et al., 2013). The cerebellum thus controls the gamma-band synchronization of the sensori-motor cortex during active tactile exploration in the rodent. Therefore, this decrease in the intra-cortical activity in gamma band could directly result from the contribution of the cerebellum to the sensorimotor coordination after cerebellar kainate administration during dystonia (Popa et al., 2013).

### Functional Implications

Motor disorders that are consecutive to brain dysfunction (e.g., Parkinson’s disease, dystonia, ataxia, tremors, etc.,) are associated with a wide variety of symptoms, but typically involve a set of several brain structures: somatosensory and premotor cortices, basal ganglia and cerebellum. Of these structures, the cerebellum is probably the least studied, despite the strong evidence for its involvement in a large spectrum of movement disorders: dystonia (Hubsch et al., 2013), essential tremor (Louis, 2014), Parkinson’s tremor (Helmich et al., 2011) and ataxia (Bastian, 2006). The cerebellum exhibits reciprocal connections with the motor cortex (Kelly and Strick, 2003; Proville et al., 2014) and the basal ganglia (Bostan and Strick, 2010). While the coarse anatomy and connectivity of the cerebello-thalamo-cortical pathway have been documented (Steriade, 1995), the cerebellar contribution to motor function and dysfunction remains unclear (Shakkottai et al., 2017). Still, this pathway is notably the site of functional reorganizations in the course of motor diseases (Brighina et al., 2009; Ni et al., 2010) anomalies in the cerebello-cortical pathway co-vary with the severity of the symptoms, but the nature of their contribution to the pathology remains unclear. We showed in our study that local anomalies in the cerebellum could induce sustained dystonia coupled with intra-cortical changes in oscillatory activities suggesting that the cerebellum is a gateway for changing motor circuits.

In conclusion, examination of cortical oscillatory activities in this animal model of dystonia caused by cerebellar dysfunction reveals a disruption of the coordination of neuronal activity across the cortical somatosensory/parietal network, which may underlie deficits in motor skills.

## Author Contributions

EG, IG, A-MZ and DP designed the experiments and wrote the manuscript. EG, IG, CZ, AŞ, VM and AP performed the experiments and analyzed the data.

## Conflict of Interest Statement

The authors declare that the research was conducted in the absence of any commercial or financial relationships that could be construed as a potential conflict of interest.
