# Supplementary material for: Oscillatory Cortical Activity in an Animal Model of Dystonia Caused by Cerebellar Dysfunction
Source: Front Cell Neurosci. 2018 Nov 6;12:390. doi: 10.3389/fncel.2018.00390 (PMC6232371; doi:10.3389/fncel.2018.00390)
Supplement: Supplementary file 1 [file Data_Sheet_1.docx]

***Supplementary Material***

**Oscillatory cortical activity in an animal model of dystonia caused by cerebellar dysfunction**

**Elena Laura Georgescu^1,2,#^, Ioana Antoaneta Georgescu^1,#^, Carmen Denise Mihaela Zahiu^1^, Alexandru Răzvan Șteopoaie^1^, Vlad- Petru Morozan^1^, Adrian Ștefan Pană^1^, Ana-Maria Zăgrean^1,^* and Daniela Popa^1, 2,^***

^1^Division of Physiology and Neuroscience, Carol Davila University of Medicine and Pharmacy, Bucharest, Romania

^2^Institut de biologie de l'Ecole normale supérieure (IBENS), Ecole normale supérieure, CNRS, INSERM, PSL Research University, 75005 Paris, France

^#^ These authors contributed equally to this work.

*These authors jointly directed this work.

*** Correspondence should be addressed to D.P (daniela.popa@ens.fr) or A.M.Z (ana-maria.zagrean@umfcd.ro)**

# Supplementary Figures

## Supplementary Figure 1:

**Supplementary Figure 2:**

**Supplementary Figure 3:**

**Supplementary Figure 4:**

**Supplementary Figure 5:**

**Supplementary Figure 6:**

**Supplementary Figure 1.** Imaginary motor-somatosensory and motor-parietal coherences during day 5 of dystonia (pre and post kainate). **(A)** Imaginary motor-somatosensory coherence for each frequency band (represented as percentage from baseline day), baseline vs day 5 (pre and post). **(B)** Imaginary motor-parietal coherence for each frequency band (represented as percentage from baseline day), baseline vs day 5 (pre and post). **(C)** Imaginary motor-somatosensory coherence across all frequencies (0,5-100 Hz), baseline vs day 5 (pre and post). **(D)** Imaginary motor-parietal coherence across all frequencies (0,5-100 Hz), baseline vs day 5 (pre and post) of intracerebellar kainic acid administration.

**Supplementary Figure 2.** Correlations between motor cortex power spectral density and behavior on day 1. **(A)** Linear regression of dystonic behavior versus motor cortex (delta, theta, beta, low gamma, high gamma) power spectral density. **(B, C)** Linear regression of AW% - pre **(B)** and post **(C)** kainic acid administration versus motor cortex (delta, theta, beta, low gamma, high gamma) power spectral density. Each point corresponds to one animal (n = 20). The plain and dotted curves correspond to the 95% tolerance and confidence bands of the linear regression, respectively.

**Supplementary Figure 3.** Correlations between somatosensory cortex power spectral density and behavior on day 1. **(A)** Linear regression of dystonic behavior versus somatosensory cortex (delta, theta, beta, low gamma, high gamma) power spectral density. **(B, C)** Linear regression of AW% - pre **(B)** and post **(C)** kainic acid administration versus somatosensory cortex (delta, theta, beta, low gamma, high gamma) power spectral density. Each point corresponds to one animal (n = 10). The plain and dotted curves correspond to the 95% tolerance and confidence bands of the linear regression, respectively.

**Supplementary Figure 4.** Correlations between parietal cortex power spectral density and behavior on day 1. **(A)** Linear regression of dystonic behavior versus parietal cortex (delta, theta, beta, low gamma, high gamma) power spectral density. **(B, C)** Linear regression of AW% – pre **(B)** and post **(C)** kainic acid administration versus parietal cortex (delta, theta, beta, low gamma, high gamma) power spectral density. Each point corresponds to one animal (n = 10). The plain and dotted curves correspond to the 95% tolerance and confidence bands of the linear regression, respectively.

**Supplementary Figure 5.** Correlations between motor-somatosensory coherence and behavior on day 1. **(A)** Linear regression of dystonic behavior versus motor-somatosensory coherence (delta, theta, beta, low gamma, high gamma). **(B, C)** Linear regression of AW% – pre **(B)** and post **(C)** kainic acid administration versus motor-somatosensory coherence (delta, theta, beta, low gamma, high gamma). Each point corresponds to one animal (n = 10). The plain and dotted curves correspond to the 95% tolerance and confidence bands of the linear regression, respectively.

**Supplementary Figure 6.** Correlations between motor-parietal coherence and behavior on day 1. **(A)** Linear regression of dystonic behavior versus motor-parietal coherence (delta, theta, beta, low gamma, high gamma). **(B, C)** Linear regression of AW% - pre **(B)** and post **(C)** kainic acid administration versus motor-parietal coherence (delta, theta, beta, low gamma, high gamma). Each point corresponds to one animal (n = 10). The plain and dotted curves correspond to the 95% tolerance and confidence bands of the linear regression, respectively.

# Supplementary Tables

**Supplementary Table 1**. Pearson correlation coefficients (r) and significance (P) values for linear regressions between motor cortex power spectral density and AW% before (pre) and after (post) kainic acid administration, *p < 0.05, ** p < 0.01, *** p < 0.001:

| Frequency band | Day 1 Pre | Day 1 Post | Day 2 Pre | Day 2 Post | Day 3 Pre | Day 3 Post | Day 4 Pre | Day 4 Post | Day 5 Pre | Day 5 Post |
| --- | --- | --- | --- | --- | --- | --- | --- | --- | --- | --- |
| delta | r =0.2993  P =0.2275 | r =0.2465  P =0.2947 | r =0.002414  P =0.9924 | r =0.2116  P =0.3845 | r =0.4271  P =0.1123 | r =-0.09952  P =0.7350 | r =0.2810  P =0.3762 | r =0.7479  P =0.0021** | r =0.5197  P =0.0568 | r =0.5109  P =0.1083 |
| theta | r =0.1024  P =0.6859 | r =0.1162  P =0.6257 | r =0.2683  P =0.2818 | r =0.1279  P =0.6019 | r =0.2310  P =0.4075 | r =-0.1983  P =0.4968 | r =0.01507  P =0.9629 | r =0.4439  P =0.1118 | r =0.5107  P =0.0620 | r =0.3858  P =0.2412 |
| beta | r=0.003306  P =0.9896 | r =0.04266  P =0.8583 | r =0.1330  P =0.5988 | r =-0.06094  P =0.8043 | r =-0.08167  P =0.7723 | r =-0.3368  P =0.2390 | r =-0.1513  P =0.6389 | r =0.2312  P =0.4265 | r =0.1746  P =0.5505 | r=0.08045  P =0.8141 |
| low gamma | r =-0.1167  P =0.6446 | r =-0.02011  P =0.9329 | r =0.09857  P =0.6972 | r =-0.1214  P =0.6205 | r =0.1234  P =0.6612 | r =-0.1533  P =0.6008 | r =-0.2810  P =0.3762 | r =0.1050  P =0.7209 | r =-0.03802  P =0.8973 | r =0.05094  P =0.8818 |
| high gamma | r =-0.1469  P =0.5607 | r =0.07482  P =0.7539 | r =0.2318  P =0.3546 | r =0.1374  P =0.5747 | r =0.2341  P =0.4011 | r =0.2261  P =0.4370 | r =-0.04357  P =0.8930 | r =0.2727  P =0.3456 | r =0.09397  P =0.7493 | r =0.2051  P =0.5452 |

**Supplementary Table 2.** Pearson correlation coefficients (r) and significance (P) values for linear regressions between motor cortex power spectral density in different frequency bands and dystonia score, post kainic acid administration:

| Frequency band | Day 1 Post | Day 2 Post | Day 3 Post | Day 4 Post | Day 5 Post |
| --- | --- | --- | --- | --- | --- |
| delta | r = -0.1103  P = 0.6435 | r =0.2961  P =0.2184 | r =0.2968  P =0.3028 | r =-0.3015  P =0.2949 | r =-0.1881  P =0.5583 |
| theta | r =-0.1469  P =0.5364 | r =0.2591  P =0.2842 | r =0.3413  P =0.2323 | r =-0.07213  P =0.8064 | r =0.02389  P =0.9412 |
| beta | r =-0.2040  P =0.3883 | r =0.2099  P =0.3885 | r =0.3404  P =0.2336 | r =-0.05723  P =0.8459 | r =0.1360  P =0.6734 |
| low gamma | r =-0.3567  P =0.1227 | r =0.1321  P =0.5899 | r =0.1767  P =0.5456 | r =-0.2623  P =0.3649 | r =0.1314  P =0.6840 |
| high gamma | r =-0.5093  P =0.0218* | r =-0.1148  P =0.6397 | r =-0.2500  P =0.3887 | r =-0.5366  P =0.0479* | r =0.02241  P =0.9449 |

**Supplementary Table 3.** Pearson correlation coefficients (r) and significance (P) values for linear regressions between somatosensory cortex power spectral density and AW% before (pre) and after (post) kainic acid administration, *p < 0.05, ** p < 0.01, *** p < 0.001:

| Frequency band | Day 1 Pre | Day 1 Post | Day 2 Pre | Day 2 Post | Day 3 Pre | Day 3 Post | Day 4 Pre | Day 4 Post | Day 5 Pre | Day 5 Post |
| --- | --- | --- | --- | --- | --- | --- | --- | --- | --- | --- |
| delta | r =0.3510  P =0.4402 | r =0.1632  P =0.6748 | r =0.7781  P =0.0684 | r =0.4150  P =0.2667 | r =0.7860  P =0.0361* | r =0.3144  P =0.4922 | r =0.3074  P =0.6149 | r =0.8060  P =0.0286* | r =0.8532  P =0.0146* | r =0.8498  P =0.3535 |
| theta | r =0.1411  P =0.7629 | r =0.06866  P =0.8607 | r =0.6798  P =0.1374 | r =0.1619  P =0.6772 | r =0.5785  P =0.1736 | r =0.5502  P =0.2007 | r =0.3485  P =0.5654 | r =0.4525  P =0.3079 | r =0.6074  P =0.1480 | r =0.01350  P =0.9914 |
| beta | r =0.3390  P =0.4570 | r =0.1781  P =0.6466 | r =0.7758  P =0.0698 | r =0.3400  P =0.3707 | r =0.6754  P =0.0959 | r =0.7404  P =0.0570 | r =0.3673  P =0.5431 | r =0.7051  P =0.0768 | r =0.5995  P =0.1548 | r =0.1919  P =0.8771 |
| low gamma | r =0.3232  P =0.4795 | r =0.2961  P =0.4392 | r =0.6725  P =0.1434 | r =0.6052  P =0.0842 | r =0.7119  P =0.0727 | r =0.6805  P =0.0925 | r =0.2597  P =0.6731 | r =0.5472  P =0.2036 | r =0.4559  P =0.3038 | r =0.3832  P =0.7496 |
| high gamma | r =0.4115  P =0.3590 | r =0.2861  P =0.4554 | r =0.7578  P =0.0809 | r =0.6525  P =0.0568 | r =0.6907  P =0.0857 | r =0.4992  P =0.2541 | r =0.2848  P =0.6424 | r =0.5274  P =0.2238 | r =0.6154  P =0.1413 | r =0.4607  P =0.6952 |

**Supplementary Table 4.** Pearson correlation coefficients (r) and significance (P) values for linear regressions between somatosensory cortex power spectral density in different frequency bands and dystonia score, post kainic acid administration:

| Frequency band | Day 1 Post | Day 2 Post | Day 3 Post | Day 4 Post | Day 5 Post |
| --- | --- | --- | --- | --- | --- |
| delta | r =-0.2192  P =0.5710 | r =-0.4167  P =0.3044 | r =-0.7398  P =0.0573 | r =-0.5045  P =0.2482 | r =-0.1178  P =0.8241 |
| theta | r =-0.2713  P =0.4801 | r =-0.5816  P =0.1305 | r =-0.6549  P =0.1104 | r =-0.4567  P =0.3030 | r =0.07185  P =0.8924 |
| beta | r =-0.3371  P =0.3751 | r =-0.6494  P =0.0814 | r =-0.7732  P =0.0414* | r =-0.6720  P =0.0982 | r =0.07073  P =0.8941 |
| low gamma | r =-0.3975  P =0.2893 | r =-0.5931  P =0.1212 | r =-0.7628  P =0.0461* | r =-0.8226  P =0.0231* | r =-0.07961  P =0.8808 |
| high gamma | r =-0.3848  P =0.3065 | r =-0.6105  P =0.1079 | r =-0.6912  P =0.0855 | r =-0.8673  P =0.0115* | r =-0.07878  P =0.8821 |

**Supplementary Table 5.** Pearson correlation coefficients (r) and significance (P) values for linear regressions between parietal cortex power spectral density and AW% before (pre) and after (post) kainic acid administration, *p < 0.05, ** p < 0.01, *** p < 0.001:

| Frequency band | Day 1 Pre | Day 1 Post | Day 2 Pre | Day 2 Post | Day 3 Pre | Day 3 Post | Day 4 Pre | Day 4 Post | Day 5 Pre | Day 5 Post |
| --- | --- | --- | --- | --- | --- | --- | --- | --- | --- | --- |
| delta | r =0.1857  P =0.6075 | r =0.7150  P =0.0201* | r =0.3093  P =0.4559 | r =0.1802  P =0.6427 | r =-0.02652  P =0.9503 | r =-0.3539  P =0.4913 | r =-0.5007  P =0.3117 | r =0.4875  P =0.2671 | r =-0.01143  P =0.9806 | r =0.6897  P =0.0864 |
| theta | r =0.3104  P =0.3828 | r =0.3036  P =0.3938 | r =-0.3688  P =0.3686 | r =0.09026  P =0.8174 | r =0.3563  P =0.3863 | r =-0.3991  P =0.4332 | r =-0.2317  P =0.6586 | r =0.1855  P =0.6904 | r =0.1227  P =0.7933 | r =0.08273  P =0.8600 |
| beta | r =0.1874  P =0.6042 | r =0.4560  P =0.1853 | r =-0.3156  P =0.4464 | r =0.1179  P =0.7626 | r =0.07205  P =0.8654 | r =-0.4902  P =0.3236 | r =-0.06726  P =0.8993 | r =0.1387  P =0.7668 | r =-0.1340  P =0.7745 | r =-0.08120  P =0.8626 |
| low gamma | r =0.1451  P =0.6891 | r =0.007926  P =0.9827 | r =-0.2768  P =0.5069 | r =0.05622  P =0.8858 | r =0.2692  P =0.5192 | r =-0.3563  P =0.4881 | r =-0.1513  P =0.7748 | r =0.2731  P =0.5534 | r =0.04603  P =0.9219 | r =0.09267  P =0.8434 |
| high gamma | r =0.4205  P =0.2263 | r =-0.1202  P =0.7409 | r =0.03699  P =0.9307 | r =0.007358  P =0.9850 | r =0.3025  P =0.4664 | r =-0.007638  P =0.9885 | r =-0.009019  P =0.9865 | r =0.3146  P =0.4920 | r=0.007879  P =0.9866 | r =0.2608  P =0.5721 |

**Supplementary Table 6.** Pearson correlation coefficients (r) and significance (P) values for linear regressions between parietal cortex power spectral density in different frequency bands and dystonia score, post kainic acid administration:

| Frequency band | Day 1 Post | Day 2 Post | Day 3 Post | Day 4 Post | Day 5 Post |
| --- | --- | --- | --- | --- | --- |
| delta | r =0.004690  P =0.9897 | r =0.4101  P =0.2729 | r =0.9829  P =0.0004*** | r =0.6046  P =0.1504 | r =0.07799  P =0.8680 |
| theta | r =-0.2972  P =0.4043 | r =0.3668  P =0.3315 | r =0.3190  P =0.5378 | r =0.3893  P =0.3880 | r =0.4981  P =0.2553 |
| beta | r =-0.4610  P =0.1800 | r =0.1993  P =0.6072 | r =0.4058  P =0.4247 | r =0.5841  P =0.1685 | r =0.4616  P =0.2971 |
| low gamma | r =-0.03547  P =0.9225 | r =0.1314  P =0.7361 | r =0.4678  P =0.3495 | r =0.4927  P =0.2612 | r =0.5942  P =0.1595 |
| high gamma | r =0.1638  P =0.6512 | r =-0.09827  P =0.8014 | r =0.4560  P =0.3634 | r =0.07291  P =0.8765 | r =0.6398  P =0.1217 |

**Supplementary Table 7**. Pearson correlation coefficients (r) and significance (P) values for linear regressions between motor-somatosensory coherence and AW% before (pre) and after (post) kainic acid administration, *p < 0.05, ** p < 0.01, *** p < 0.001:

| Frequency band | Day 1 Pre | Day 1 Post | Day 2 Pre | Day 2 Post | Day 3 Pre | Day 3 Post | Day 4 Pre | Day 4 Post | Day 5 Pre | Day 5 Post |
| --- | --- | --- | --- | --- | --- | --- | --- | --- | --- | --- |
| delta | r = 0.4874  P = 0.2206 | r = -0.6884  P = 0.0277 * | r = -0.6542  P = 0.0559 | r = -0.7777  P = 0.0231 * | r = -0.3828  P = 0.3967 | r = -0.3010  P = 0.4312 | r = -0.2608  P = 0.5722 | r = -0.5541  P = 0.1542 | r = -0.5166  P = 0.2351 | r = -0.9324  P = 0.0676 |
| theta | r = 0.5139  P = 0.1927 | r = -0.6773  P = 0.0314 * | r = -0.3676  P = 0.3304 | r = -0.7544  P = 0.0306 * | r = 0.09419  P = 0.8408 | r = -0.6243  P = 0.0723 | r = -0.1097  P = 0.8149 | r = -0.4656  P = 0.2449 | r = -0.1095  P = 0.8152 | r = -0.5487  P = 0.4513 |
| beta | r = 0.4908  P = 0.2169 | r = -0.5177  P = 0.1254 | r = -0.5855  P = 0.0976 | r = -0.7645  P = 0.0271 * | r = -0.1739  P = 0.7092 | r = -0.5803  P = 0.1014 | r = -0.3268  P = 0.4743 | r = -0.5386  P = 0.1684 | r = -0.3935  P = 0.3824 | r = -0.7883  P = 0.2117 |
| low gamma | r = 0.5167  P = 0.1898 | r = -0.3788  P = 0.2804 | r = -0.5588  P = 0.1178 | r = -0.7286  P = 0.0404 * | r = -0.09325  P = 0.8424 | r = -0.4998  P = 0.1707 | r = -0.6230  P = 0.1351 | r = -0.6302  P = 0.0940 | r = -0.6166  P = 0.1403 | r = -0.8520  P = 0.1480 |
| high gamma | r = 0.7005  P = 0.0530 | r = -0.3985  P = 0.2540 | r = -0.1996  P = 0.6066 | r = -0.5637  P = 0.1456 | r = 0.3163  P = 0.4895 | r = -0.4568  P = 0.2164 | r = -0.5234  P = 0.2280 | r = -0.5652  P = 0.1443 | r = -0.3598  P = 0.4279 | r = -0.5012  P = 0.4988 |

**Supplementary Table 8.** Pearson correlation coefficients (r) and significance (P) values for linear regressions between motor-somatosensory coherence in different frequency bands and dystonia score, post kainic acid administration:

| Frequency band | Day 1 Post | Day 2 Post | Day 3 Post | Day 4 Post | Day 5 Post |
| --- | --- | --- | --- | --- | --- |
| delta | r = 0.07503  P = 0.8368 | r = 0.6420  P = 0.0861 | r = 0.3465  P = 0.3610 | r = 0.6023  P = 0.1141 | r = 0.2131  P = 0.6853 |
| theta | r = 0.2735  P = 0.4445 | r = 0.5260  P = 0.1806 | r = 0.03721  P = 0.9243 | r = 0.4841  P = 0.2242 | r = -0.3621  P = 0.4805 |
| beta | r = 0.1838  P = 0.6113 | r = 0.6399  P = 0.0875 | r = -0.02778  P = 0.9434 | r = 0.4628  P = 0.2482 | r = -0.3901  P = 0.4445 |
| low gamma | r = 0.06834  P = 0.8512 | r = 0.6197  P = 0.1013 | r = 0.05091  P = 0.8965 | r = 0.4071  P = 0.3168 | r = -0.1981  P = 0.7067 |
| high gamma | r = -0.002965  P = 0.9935 | r = 0.3910  P = 0.3381 | r = 0.04103  P = 0.9165 | r = 0.3286  P = 0.4268 | r = -0.1369  P = 0.7960 |

**Supplementary Table 9.** Pearson correlation coefficients (r) and significance (P) values for linear regressions between motor-parietal coherence and AW% before (pre) and after (post) kainic acid administration, *p < 0.05, ** p < 0.01, *** p < 0.001:

| Frequency band | Day 1 Pre | Day 1 Post | Day 2 Pre | Day 2 Post | Day 3 Pre | Day 3 Post | Day 4 Pre | Day 4 Post | Day 5 Pre | Day 5 Post |
| --- | --- | --- | --- | --- | --- | --- | --- | --- | --- | --- |
| delta | r =-0.06028  P =0.8686 | r =-0.05009  P =0.8907 | r =-0.3873  P =0.3031 | r =-0.3425  P =0.3669 | r =-0.2807  P =0.5420 | r =-0.02422  P =0.9589 | r =-0.6136  P =0.1428 | r =0.03912  P =0.9267 | r =-0.1754  P =0.6778 | r =0.3251  P =0.4321 |
| theta | r =0.1391  P =0.7015 | r =-0.2397  P =0.5047 | r =-0.4339  P =0.2433 | r =-0.5571  P =0.1192 | r =0.1583  P =0.7346 | r =0.2819  P =0.5402 | r =-0.1902  P =0.6830 | r =-0.03828  P =0.9283 | r =-0.01645  P =0.9692 | r =0.2791  P =0.5033 |
| beta | r =0.1811  P =0.6165 | r =0.07556  P =0.8357 | r =-0.3738  P =0.3217 | r =-0.4894  P =0.1812 | r =0.3354  P =0.4621 | r =0.4194  P =0.3489 | r =0.05309  P =0.9100 | r =0.06304  P =0.8821 | r =0.04449  P =0.9167 | r =0.3055  P =0.4619 |
| low gamma | r =0.2149  P =0.5509 | r =0.03615  P =0.9210 | r =-0.3160  P =0.4074 | r =-0.5186  P =0.1526 | r =0.4465  P =0.3152 | r =0.2892  P =0.5293 | r =-0.05435  P =0.9079 | r =0.1587  P =0.7074 | r =0.2020  P =0.6315 | r =0.4336  P =0.2831 |
| high gamma | r =0.2646  P =0.4601 | r =-0.1499  P =0.6795 | r =-0.3903  P =0.2991 | r =-0.5341  P =0.1386 | r =0.3086  P =0.5007 | r =0.1066  P =0.8200 | r =-0.07857  P =0.8670 | r =-0.08705  P =0.8376 | r =-0.07712  P =0.8560 | r =0.09966  P =0.8144 |

**Supplementary Table 10.** Pearson correlation coefficients (r) and significance (P) values for linear regressions between motor-parietal coherence in different frequency bands and dystonia score, post kainic acid administration:

| Frequency band | Day 1 Post | Day 2 Post | Day 3 Post | Day 4 Post | Day 5 Post |
| --- | --- | --- | --- | --- | --- |
| delta | r =-0.5595  P =0.0926 | r =0.7036  P =0.0344* | r =0.2518  P =0.5859 | r =0.1470  P =0.7283 | r =0.04318  P =0.9191 |
| theta | r =-0.4626  P =0.1783 | r =0.2672  P =0.4871 | r =-0.4367  P =0.3273 | r =0.2140  P =0.6108 | r =0.2492  P =0.5518 |
| beta | r =-0.5806  P =0.0784 | r =0.3379  P =0.3739 | r =-0.2719  P =0.5552 | r =0.1773  P =0.6745 | r =0.05442  P =0.8982 |
| low gamma | r =-0.5701  P =0.0853 | r =0.3032  P =0.4278 | r =-0.1614  P =0.7296 | r =0.1525  P =0.7185 | r =0.01571  P =0.9706 |
| high gamma | r =-0.5551  P =0.0958 | r =0.3522  P =0.3526 | r =-0.4653  P =0.2928 | r =0.09464  P =0.8236 | r =0.1925  P =0.6478 |
